# Genomic insights into host adaptation between the wheat stripe rust pathogen (*Puccinia striiformis* f. sp. *tritici*) and the barley stripe rust pathogen (*Puccinia striiformis* f. sp. *hordei*)

**DOI:** 10.1186/s12864-018-5041-y

**Published:** 2018-09-12

**Authors:** Chongjing Xia, Meinan Wang, Chuntao Yin, Omar E. Cornejo, Scot H. Hulbert, Xianming Chen

**Affiliations:** 1 Department of Plant Pathology, Washington State University, Pullman, WA 99164-6430 USA; 2 School of Biological Sciences, Washington State University, Pullman, WA 99164-7520 USA; 3 Wheat Health, Genetics, and Quality Research Unit, Agriculture Research Service, U.S. Department of Agriculture, Pullman, WA 99164-6430 USA

**Keywords:** Stripe rust, *Puccinia striiformis*, Formae speciales, Host adaptation, Comparative genomics, Gene loss, Evolution

## Abstract

**Background:**

Plant fungal pathogens can rapidly evolve and adapt to new environmental conditions in response to sudden changes of host populations in agro-ecosystems. However, the genomic basis of their host adaptation, especially at the *forma specialis* level, remains unclear.

**Results:**

We sequenced two isolates each representing *Puccinia striiformis* f. sp. *tritici* (*Pst*) and *P. striiformis* f. sp. *hordei* (*Psh*), different formae speciales of the stripe rust fungus *P. striiformis* highly adapted to wheat and barley, respectively. The divergence of *Pst* and *Psh*, estimated to start 8.12 million years ago, has been driven by high nucleotide mutation rates. The high genomic variation within dikaryotic urediniospores of *P. striiformis* has provided raw genetic materials for genome evolution. No specific gene families have enriched in either isolate, but extensive gene loss events have occurred in both *Pst* and *Psh* after the divergence from their most recent common ancestor. A large number of isolate-specific genes were identified, with unique genomic features compared to the conserved genes, including 1) significantly shorter in length; 2) significantly less expressed; 3) significantly closer to transposable elements; and 4) redundant in pathways. The presence of specific genes in one isolate (or forma specialis) was resulted from the loss of the homologues in the other isolate (or forma specialis) by the replacements of transposable elements or losses of genomic fragments. In addition, different patterns and numbers of telomeric repeats were observed between the isolates.

**Conclusions:**

Host adaptation of *P. striiformis* at the forma specialis level is a complex pathogenic trait, involving not only virulence-related genes but also other genes. Gene loss, which might be adaptive and driven by transposable element activities, provides genomic basis for host adaptation of different formae speciales of *P. striiformis*.

**Electronic supplementary material:**

The online version of this article (10.1186/s12864-018-5041-y) contains supplementary material, which is available to authorized users.

## Background

Filamentous plant pathogens (e.g. fungi and oomycetes) cause severe diseases of crops and threaten global food security [1]. Remarkably, the populations of many devastating pathogens have been rapidly evolving and continue to emerge or reemerge in the agrio-ecosystems [2]. Understanding their evolution will be valuable for monitoring such pathogens, and for designing strategies and methods for sustainable management. The advance of whole-genome sequencing technologies has enabled us to disentangle the genomic basis underlying the host-pathogen co-evolution and demonstrated that the ‘two-speed genomes’ have contributed to the rapid evolution of filamentous plant pathogens [3–5]. In the ‘two-speed genome’ concept, genome parts (or genomic compartments) harboring pathogenicity-related genes, which are usually conditionally dispensable, have a higher evolution rate than the essential parts of the genome. Tremendous genomic studies of filamentous plant pathogens have shown that the pathogenicity-related genes reside in such rapidly evolving genomic compartments including gene clusters [6], gene-sparse and repeat-rich regions [7], AT-rich isochores [8] and supernumerary (conditionally dispensable) chromosomes [9, 10]. While most of these studies were on pathogenicity-related genes, details of genomic basis of host adaptation in filamentous plant pathogens remain to be elucidated even though some of the pathogenicity genes were also host specificity determinants [10].

Among filamentous plant pathogens, the genus *Puccinia* is a group of destructive rust fungi. As obligate biotrophs, they depend on living plant cells to complete their life cycle, and are considered highly specialized on their hosts. A single *Puccinia* species may have different forms, with each form mainly infecting specialized genera or species of susceptible hosts but not distinguishable from other forms by morphological characters. Such forms are called formae speciales (sing. forma specialis; abbr. f. sp.). For instance, *Puccinia striiformis*, causing stripe (yellow) rust diseases on cereal crops and grasses, has been reported to have nine formae speciales [11]. The two most economically important forms are *P. striiformis* f. sp. *tritici* (*Pst*) and *P. striiformis* f. sp. *hordei* (*Psh*), specialized, but not exclusively, on wheat and barley, respectively. Although their host ranges overlap on some grasses [12, 13], the two formae speciales were clearly separated into closely-related but distinct lineages based on molecular markers [13]. Formae speciales have been documented in many other devastating filamentous plant pathogen species, including *Blumeria graminis* causing powdery mildews on cereal crops and grasses [14], *Fusarium oxysporum* causing vascular wilts and blights on many plant species [15] and *Sphacelotheca reiliana* (also known as *Sporisorium reilianum*) causing head smuts of maize, sorghum and related plants [16]. Genomics studies have begun to shed light on our understanding of formae speciales. For example in *B. graminis,* comparative genomics determined that the forma specialis *triticale*, which is pathogenic on triticale derived from crosses between *Triticum* and *Secale* species, is a hybrid of two other formae speciales *tritici* and *secalis* [17]. However, in most cases the evolution of formae speciales within a species remains obscure. Only a few isolates of *Pst* but no isolates of *Psh* have been sequenced, and therefore, it was not clear whether *Pst* and *Psh* have divergent genomes or how they evolved into different formae speciales.

The objective of this study was to decipher the genomic basis of host adaptation of *P. striiformis* at the forma specialis level. We selected two *P. striiformis* isolates to represent formae speciales *tritici* and *hordei*, highly adapted to wheat and barley, respectively. We took the advantages of advanced sequencing technologies to generate reference genomes with high continuity for comparative analysis. Our data suggest that the rapidly evolving and loss-of-function genes provide the potential genomic basis for host adaption in *P. striiformis*.

## Results

### Host adaptation of *P. striiformis*

**Fig. 1:**
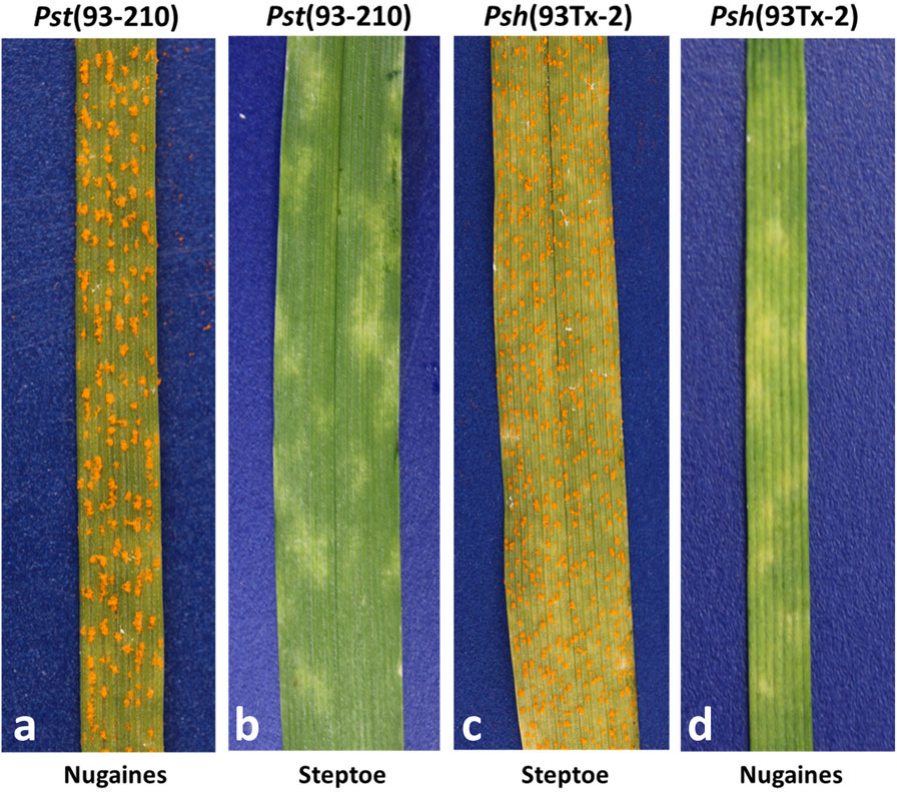
Host adaptation of *Puccinia striiformis*. **a**, **b** Pathogenicity test of *Pst* (93–210) on wheat cultivar Nugaines and barley cultivar Steptoe. **c**, **d** Pathogenicity test of *Psh* (93TX-2) on barley cultivar Steptoe and wheat cultivar Nugaines. Note that wheat cultivar Nugaines is susceptible to all *Pst* isolates collected in the U.S., and barley cultivar Steptoe is susceptible to all *Psh* isolates collected in the U.S

We selected isolates 93–210 of *Pst* and 93TX-2 of *Psh* to represent the wheat and barley stripe rust fungi, respectively for understanding the potential genomic basis of their host adaptation. Our pathogenicity tests showed that the *Pst* (93–210) and *Psh* (93TX-2) isolates were highly virulent to their respective hosts, but highly avirulent to the other host (Fig. 1). Moreover, *Pst* (93–210) was virulent to wheat lines with stripe rust resistance genes *Yr2*, *Yr17*, *Yr21* and *Yr28*, but avirulent to all barley stripe rust differential cultivars. Similarly, *Psh* (93TX-2) was virulent to barley differential cultivars Topper, Emir and Abed Binder 12, but avirulent to all wheat differential lines. In summary, our pathogenicity tests showed the host adaptations of these two isolates, and confirmed their status of different formae speciales of *P. striiformis*.

### Genome features and comparison

We sequenced isolates *Pst* (93–210) and *Psh* (93TX-2) using both Illumina HiSeq PE150 (> 70-fold coverage) and the Pacific Biosciences (PacBio) single molecule real-time sequencing technology (> 50-fold coverage). This is the first report for the genome sequence of *Psh*, the barley stripe rust pathogen. Both isolates were predicted to be approximately 89 Mb in genome size and highly heterozygous (Table 1; Additional file 1: Figure S1). Both genomes contain high but slightly different percentages of repeated sequences, 36.03% in *Pst* (93–210) and 34.20% in *Psh* (93TX-2), which are slightly higher than those of isolates 2K-41-Yr9 (PST-78) and CYR32 but lower than that of isolate 104E 137A- [18–23]. Differences in the presence and number of unique transposable elements were observed between *Pst* (93–210) and *Psh* (93TX-2) and also between these and other *Pst* isolates (Additional file 2: Table SE1) [18–23].

**Table 1:** Genome statistics of *Puccinia striiformis* isolates *Pst* (93–210) and *Psh* (93TX-2) in comparison with previously sequenced isolates^a^

	*Pst* (93–210)	*Psh* (93TX-2)	*Pst* 104E 137A-	*Pst* 2 K-41-Yr9	*Pst*-CY32
Assembly statistics					
Genome size in contigs (bp)	84,627,802	77,368,122	83,355,616	79,310,008	115,495,157
GC-content (%)	44.39	44.40	44.40	44.43	44.76
Number of contigs	493	562	156	17,295	10,913
Number of contigs > 100 kb	243	263	93	3	25
Contig N50	83	110	23	1320	1434
Contig N50 length (bp)	295,440	218,468	1,304,018	17,361	21,648
Mean contig size (bp)	171,659	137,666	534,331	4586	10,583
Annotation statistics					
Protein-coding genes	16,513	15,976	15,928	19,912	25288^b^
Annotation completeness^c^ (%)	95.10	93.00	97.60	98.60	94^b^
Secreted proteins	1517	1624	2059	2056	2092^b^
tRNA genes	648	626	636	668	862
Transposable elements (Mb)	30.46	26.41	31.08	24.87	37.68
Transposable elements (%)	36.03	34.2	37.28	31.35	32.62

^a^Statistics were calculated using the latest references and annotations except noted elsewhere

^b^Data was directly retrieved from Zheng et al. (2013) as the annotation of *Pst*-CY32 was not publicly available at the time of writing this paper

^c^Program BUSCO [95] was used to evaluate the annotation completeness of assembled genomes

We predicted 16,513 and 15,976 protein-coding genes in *Pst* (93–210) and *Psh* (93TX-2), respectively. Among them, 46.26 and 45.68% were validated by the RNA sequences of these isolates obtained in this study. The annotations of the two genomes are similar in completeness, at 95.10% for *Pst* (93–210) and 93.00% for *Psh* (93TX-2), which are comparable to previously annotated *Pst* genomes (Additional file 3: Table S1) [18–23]. The *Psh* (93TX-2) genome encodes 1624 (10.96%) predicted secreted proteins (SPs), slightly higher than the number from *Pst* (93–210) (1517, or 9.18%) but within the range predicted for other *Pst* isolates (8.27 to 12.92%) (Table 1). Our blastp and domain-based searches functionally annotated approximately 50% of the predicted genes: (1) 8151 and 7776 were mapped to at least one gene ontology (GO) term in *Pst* (93–210) and *Psh* (93TX-2) genomes, respectively; (2) 6450 and 6038 were found to have functionally defined homologues in the Pfam database for the two isolates, respectively; and (3) 1672 and 1131 were mapped to enzymes in the Kyoto Encyclopedia of Genes and Genomes (KEGG) database, respectively. No particular GO terms were significantly enriched in either genome. Comparisons of selected protein families also did not show apparent differences between *Pst* and *Psh*, and were also not found when wheat or barley adapted forma speciales of *B. graminis* were compared (Table 2; Additional file 1: Figure S2; Additional file 2: Table SE2–8) [24, 25].

**Table 2:** Comparison of specific protein families of *Puccinia striiformis* isolates *Pst* (93–210) and *Psh* (93TX-2) with *Blumaria graminis* isolates *Bgt* and *Bgh*^a^

	*Pst* (93–210)	*Psh* (93TX-2)	*Bgt*	*Bgh*
CAZymes	369	349	182	184
Cytochrome P450	21	19	10	9
Genes involved in pathogen-host interaction	748	693	1059	1030
Hydrolases	878	807	564	594
Isomerases	59	63	52	57
Ligases	87	89	73	78
Lyases	68	56	59	60
Mating-related	7	4	4	3
Oxidoreductases	192	177	170	174
Proteases	1160	1056	989	1025
Secondary metabolites	29	33	18	18
Transcription factors	104	88	73	76
Transferases	427	397	376	386
Transporters	407	381	466	407

^a^The data of *Pst* (93–210) and *Psh* (93TX-2) were generated in this study and the data of *Bgt* and *Bgh* were from Wicker et al. 2013 and Spanu et al. 2010

**Fig. 2:**
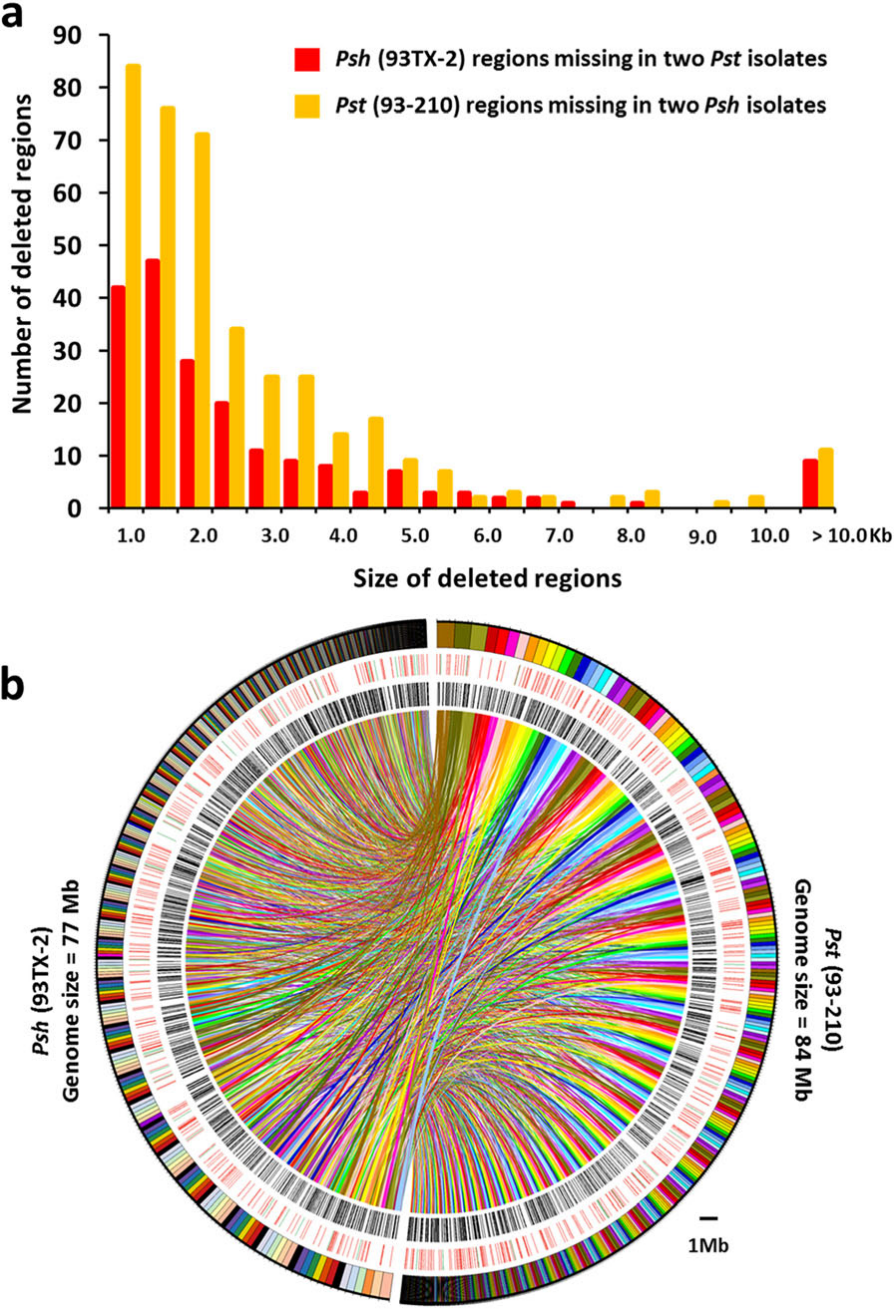
Genome comparison and synteny analysis. **a** Distributions of various sizes of deleted genome regions presented in isolate *Psh* (93TX-1) but missing in two *Pst* isolates (93–210 and 2K-41-Yr9) and those presented in isolate *Pst* (93–210) but missing in two *Psh* isolates (93TX-2 and 04–051) of *Puccinia striiformis*. **b** Genome-wide syntenic relationships between *Pst* (93–210) and *Psh* (93TX-2). The right and left in the Circos diagram are *Pst* (93–210) and *Psh* (93TX-2) genomes, respectively. Each bar in the outer layer represents one contig, organized from largest to shortest clockwise. The second layer shows genes involved in the evolution of gene families. Green and red bars are genes involved in family gain and loss, respectively. Black bars in the third layer represent genes specific to each isolate and without orthologs in the other 15 tested plant fungal pathogens. The colored links in the inner layer represent homologous gene pairs between *Pst* (93–210) and *Psh* (93TX-2)

We observed extensive large syntenic blocks of nucleotides shared among the *Pst* (93–210), *Psh* (93TX-2) and *Pst* (104E 137A-) genomes [23]. The mean lengths of syntenic blocks are 12.64 and 13.06 kb when comparing *Psh* (93TX-2) to *Pst* (93–210) and *Pst* (104E 137A-), respectively. These mean lengths are shorter (approximately 66%) than that of the syntenic blocks between the two *Pst* isolates (19.55 kb) (Additional file 1: Figure S3). Similarly, we observed slightly lower mean nucleotide identities in the *Psh*-*Pst* isolate comparisons (97.30 and 97.32%) than the mean nucleotide identity between *Pst* and *Pst* isolates (97.59%). These data suggested that isolates within a forma specialis are more closely related than isolates of different formae speciales, and therefore, the data confirmed the taxonomic relationships between *Pst* and *Psh*. By reciprocal mapping of the Illumina reads to both genomes, we did not detect large deleted regions but only mosaics of small regions (< 50 kb) that were present in one reference genome but missing in the other forma specialis (Fig. 2a; Additional file 1: Figure S4). The total lengths of form-specific regions were 988 and 495 kb, harboring 87 and 48 genes, in *Pst* (93–210) and *Psh* (93TX-2), respectively. Besides the high degree of similarity at the nucleotide level, *Pst* (93–210) and *Psh* (93TX-2) also share large collinear orthologous blocks (Fig. 2b; Additional file 1: Figure S5). We observed that only 10,769 (63.79%) of the *Pst* (93–210) predicted proteins are homologous with 10,417 (65.20%) of the *Psh* (93TX-2) proteins (see Methods).

Using the OrthoFinder program, we assigned 175,188 protein sequences (89.4% of total) to 20,696 orthogroups. There were 2160 orthogroups with all fungal species present and 452 of these consisted entirely of single-copy genes. In total, 15,641 (94.7%) of *Pst* (93–210) proteins and 15,009 (93.9%) of *Psh* (93TX-2) proteins were assigned to 9165 and 9044 orthogroups, respectively. Of these, 10,769 (63.79%) of *Pst* (93–210) proteins and 10,417 (65.20%) of *Psh* (93TX-2) proteins were homologous and assigned to 9316 orthogroups. This indicated that 4872 (15,641-10,769) of *Pst* (93–210) proteins and 4592 (15,009-10,417) of *Psh* (93TX-2) proteins had orthologues in other fungi, but not in *Psh* (93TX-2) or *Pst* (93–210), respectively*.*

In addition to nuclear genomes, we also sequenced the complete mitochondrial (mt) genomes of *Pst* (93–210) and *Psh* (93TX-2) (Additional file 1: Figure S6; Additional file 3: Tables S2-S3). No difference was observed between the *Pst* and *Psh* genomes at the genic and structural levels.

### Divergence and evolution of *Pst* and *Psh*

**Fig. 3:**
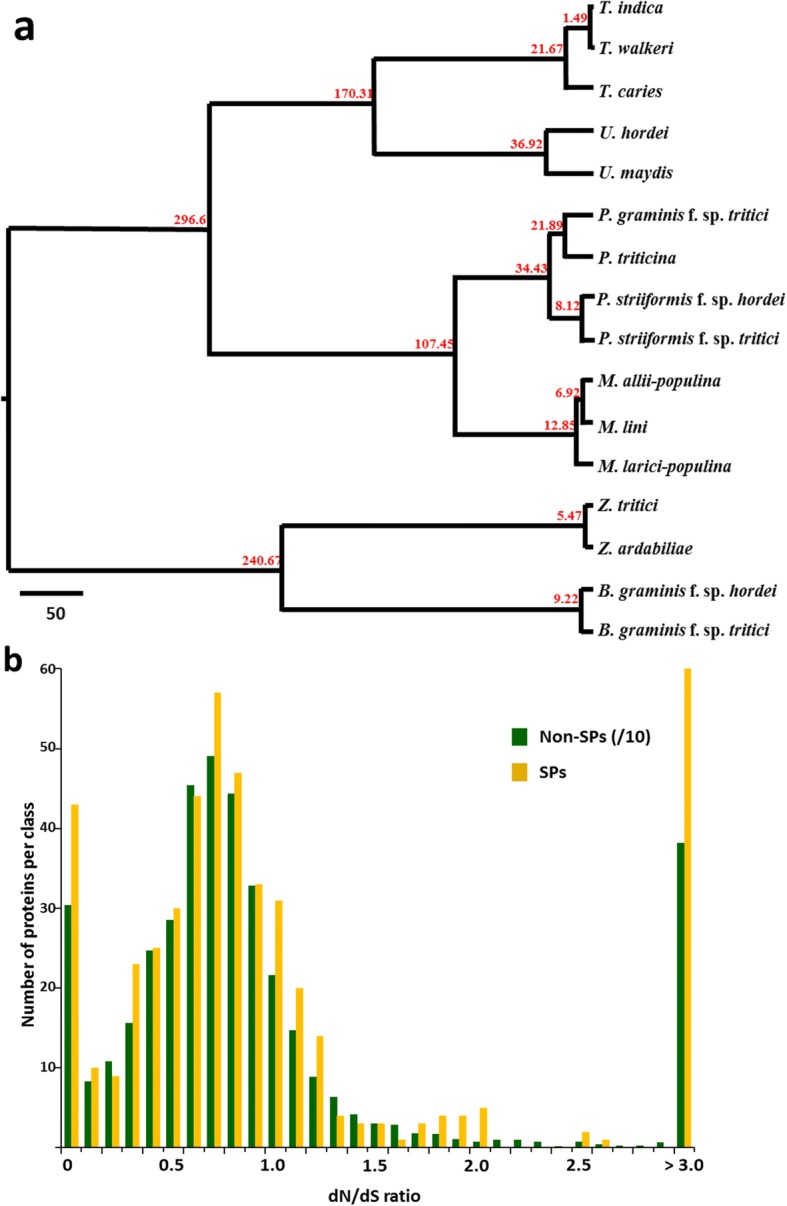
Divergence and dN/dS analysis. **a** Estimation of the divergence times of selected fungal plant pathogens. The ultrametric tree was inferred from 439 single-copy orthologs (predicted coding sequences). Divergence times were estimated using r8s and calibrated on the 452 million years divergence between Ascomycetes and Basidiomycetes. *B*: *Blumeria*; *M*: *Melampsora*; *P*: *Puccinia*; *T*: *Tilletia*; *U*: *Ustilago*; and *Z*: *Zymoseptoria*. *Z*. *tritici* traditionally called *Mycosphaerella graminicola*. **b** Comparison of 4486 pairs of *Pst* (93–210) and *Psh* (93TX-2) single-copy homologs. The X axis represents the ratio of nonsynonymous to synonymous substitutions (dN/dS) for all gene pairs, and the Y axis represents the number of gene pairs in each dN/dS range. The orange bars represent the 478 gene pairs of *Pst* (93–210) and *Psh* (93TX-2) single-copy homologs encoding secreted protein (SP) genes, and the green bars represent the 4008 non-SP-coding gene pairs. For better visibility, the numbers for non-SP genes were divided by 10

Based on the substitutions of 452 single-copy homologs in 16 selected plant pathogenic fungi, we estimated that *Pst* and *Psh* diverged approximately 8.12 million years ago (Fig. 3a), several million years after the divergence of their hosts (10–15 million years ago) [26, 27]. Our findings also indicated that the two formae speciales of *P. striiformis* were diverged around the time of the divergence of *Bgt* and *Bgh* (9.22 million years), which are also specialized on wheat and barley, respectively [24]. Based on our estimated divergence time and the substitutions of 5611 pairs of single-copy gene homologs between *Pst* (93–210) and *Psh* (93TX-2), we further estimated the nucleotide mutation rate as 2.0 × 10^− 8^ per base per year, which is ~ 40 fold higher than that of humans (0.033–0.047 × 10^− 8^) [28]. The high mutation rate could provide the ability to generate genomic variations in *P. striiformis* and accelerate adaptations to different plant hosts by its different formae speciales.

We detected a high frequency of heterozygosity between the two nuclei within each isolate at 6–7 SNPs per kb (Table 3), which is comparable to another US *Pst* isolate [19]. Reciprocal mapping of the Illumina reads to each reference showed that the inter-isolate SNP density was 1.20-fold higher than that of intra-isolate. We also observed both high intra- and inter-isolate InDel densities at ~ 2 InDels per kb. Remarkably, the functional prediction indicated that the intra-isolate mutations changed many protein-coding genes, 5909 in *Pst* (93–210) and 5659 in *Psh* (93TX-2) (Additional file 3: Tables S4 and S5). Among these, 1920 and 2147 genes were affected by SNPs in *Pst* (93–210) and *Psh* (93TX-2); and the InDels affected 5364 and 5174 genes in these isolates, respectively. The affected genes are diverse in predicted function, including transporters and transcription factors (TFs) (Additional file 3: Table S5). Among the transporters, there are two superfamilies enriched in genes impacted by mutations, the P-type ATPase superfamily [16 genes in *Pst* (93–210) and 14 genes in *Psh* (93TX-2)] and the protein kinase superfamily [17 in *Pst* (93–210) and 18 in *Psh* (93TX-2)] (Additional file 2: Table SE8). The proteins in the former superfamily are essential for ion-transporting; while some proteins in the latter superfamily are presumed to contribute to signal transduction and fungal pathogenesis. Remarkably in TFs, most of the heat shock factors [7 out of 8 in *Pst* (93–210) and 6 out of 10 in *Psh* (93TX-2)] were highly impacted by mutations, while all remaining TFs had moderate impacts (e.g., codon insertions and deletions, non-synonymous substitutions). Our data indicated that the mutations between two nuclei of a *P. striiformis* urediniospore have contributed to the intra-isolate genetic diversity and provided the evolutionary potential for host adaptation.

**Table 3:** Summary of intra- and inter-isolate variations of *Puccinia striiformis* isolates *Pst* (93–210) and *Psh* (93TX-2)

Polymorphism	*Pst* (93–210)		*Psh* (93TX-2)	
	*Pst* (93–210)	*Psh* (93TX-2)	*Pst* (93–210)	*Psh* (93TX-2)
SNPs				
Total SNPs	520,730	629,641	684,747	563,165
SNP density	6.42 ± 11.80/kb	7.71 ± 12.80/kb	9.16 ± 14.05/kb	7.58 ± 12.68/kb
InDels				
Total InDels	152,392	150,289	159,457	156,529
InDel density	1.97 ± 4.07/kb	1.93 ± 3.88/kb	2.21 ± 4.07/kb	2.19 ± 4.18/kb

We used dN/dS analysis to detect genes under positive selection during the evolution of *P. striiformis*. We did not observe significant differences (*p* = 0.92, two-tailed student t-test) between the dN/dS ratio distributions of SP and non-SP genes (0.7733 ± 0.56 and 0.7759 ± 0.62, respectively) (Fig. 3b). Among the 5611 single-copy protein genes that had homologues in *Pst* (93–210) and *Psh* (93Tx-2), 1125 were identical and 4486 were different between the two isolates. When the dN/dS ratios of the 4486 genes were compared, 28.33% (1271) had dN/dS ratios greater than 1.0, indicating positive selection. Together, these data suggested that a large number of both SP- and non-SP-coding genes have been positively selected during the *Pst* and *Psh* divergence. We found that genes under purifying selections are enriched for specific families except those with SP genes (Additional file 3: Table S5). For instance, 3.76% of the genes under purifying selections are transporter-coding genes, with only 1.25% of the genes in this family predicted to be under positive selection and this trend was observed in most other protein families. However, more SP-coding genes were under positive selection (11.48%) than those under purifying selection (9.95%). The SP genes under positive selection are involved in diverse pathways (2 in metabolic pathways, 4 in translations and protein processing in endoplasmic reticula and 2 in signal transduction).

### Loss of gene families

**Fig. 4:**
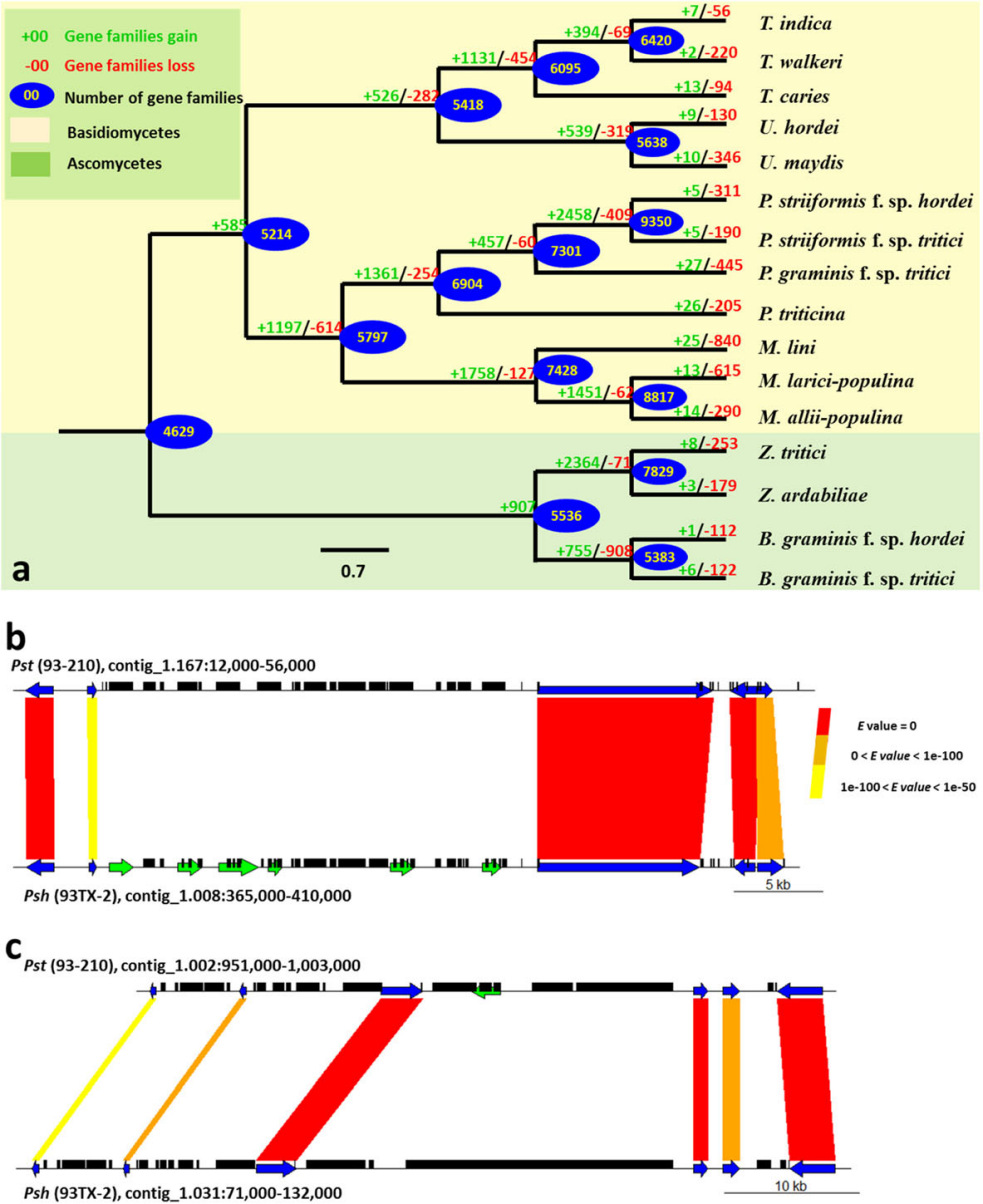
Rapidly evolving gene families in *Puccinia striiformis*. **a** Gene family evolution in 16 fungal plant pathogen genomes. Protein families were assigned using OrthoFinder. The topologies and numbers of gene family gains and losses were estimated using the DOLLOP program from PHYLIP. The positive and negative numbers on the branches represent the numbers of gene families gained and lost, respectively, during the evolution compared to the putative pan-proteome. The numbers in blue ovals correspond to the inferred ancestral protein families along each lineage. **b**, **c** Chromosomal synteny of two representative regions harboring genes involved in loss events. Blue arrows represent conserved genes, while predicted genes involved in loss events are shown in green and black blocks represent homology to known transposable element families. Yellow to red shades indicate the levels of sequence similarity based on the blast *E*-values

We examined the evolutions of the gene families of *Pst* (93–210) and *Psh* (93TX-2) in 16 plant pathogenic fungi (Additional file 2: Tables SE9 and SE10). We observed extensive gene families gained at internodes, while gene family losses were much more prominent at externodes on our reconstructed phylogenetic tree (Fig. 4a). This indicates that gene family losses have occurred during speciation or formation of formae speciales in all of the plant pathogenic fungi we examined. We noted that the topology inferred from the presence or absence of gene families (Fig. 4a) was different from that inferred from amino acid substitutions (Fig. 3a), indicating that gene family evolution was independent of substitution events. Particularly for *Pst* (93–210) and *Psh* (93TX-2), five gene families were gained in each genome after the divergence from their most recent common ancestor (Fig. 4a). The gained five gene families (containing 21 genes) in *Pst* (93–210) have unknown functions since they do not contain any Pfam domains or GO terms (Table 4). Only one gained gene in *Pst* (93–210) encodes a predicted SP. The functions of most (36 of 39 genes) of the gained five families in *Psh* (93TX-2) were also unknown. In contrast, both *Pst* (93–210) and *Psh* (93TX-2) experienced more gene family losses (190 and 311 families, respectively) after the divergence from the most recent common ancestor. Generally, *Psh* (93TX-2) experienced more extensive losses in the numbers of gene families and individual genes. In either *Pst* (93–201) or *Psh* (93Tx-2) genome, some genes encoding predicted SPs were mentioned in one of the genomes and expressed only in the genome keeping the genes based on our RNA-Seq analysis. *Psh* (93TX-2) lost more KEGG pathway-related genes (30.9%, or 141 out of 456) than *Pst* (93–210) (20.0%, or 65 out of 325) (Additional file 2: Table SE11). The GO term annotation suggested that the missing proteins are involved in diverse functions in both genomes. However, we did not detect significant enrichment in GO terms in the missing proteins compared to the rest of the proteins in both genomes. Taking together, both *Pst* (93–210) and *Psh* (93TX-2) lost many functional genes.

**Table 4:** Summary of gene family evolution in *Puccinia striiformis* isolates *Pst* (93–210) and *Psh* (93TX-2)

	*Pst* (93–210)			*Psh* (93TX-2)		
	Gain	Loss	Specific	Gain	Loss	Specific
No. of families	5	190	–	5	311	–
No. of genes	21	325	861	39	456	955
No. of SP-coding genes	1	14	61	0	34	84
No. of gene expressed	0	147	120	2	253	155
Enzymes						
Hydrolases	0	13	28	0	39	18
Isomerases	0	0	0	0	1	4
Ligases	0	4	1	0	1	8
Lyases	0	1	1	0	6	0
Oxidoreductases	0	2	6	0	8	1
Transferases	0	6	10	0	8	17
GO terms						
Total proteins with GO	0	155	147	4	268	183
Molecular function	0	73	186	4	156	229
Biological pathway	0	106	185	0	187	275
Cellular component	0	60	115	0	118	185

The genes involved in loss events are randomly dispersed along the contigs (Fig. 2b), with only few small clusters (up to six genes). As an example, one such cluster is shown in Fig. 4b, in which six genes were lost in the *Pst* (93–210) genome but present in the corresponding region in the *Psh* (93TX-2) genome. The upper panel in Fig. 4b shows that the corresponding regions of the six lost genes are replaced by transposable elements (TEs), suggesting the effects of TEs on the gene loss events. Remarkably, all these six genes with unknown functions are expressed in *Psh* (93TX-2), but not in *Pst* (93–210). Fig. 4c shows a special case in which most of the lost genes are singletons scattered in highly syntenic regions. Even so, the associations of gene losses with TE involvements are not uncommon (Fig. 4c). In some cases [161 in *Pst* (93–210) and 193 in *Psh* (93TX-2)], TE fragments are inserted in the lost genes.

### Rapidly evolving isolate-specific genes

Besides the high mutation rates and pervasive gene losses, we also noticed the existence of a large number of protein-coding genes that are species- (or forma specialis-) specific among the 16 fungal genomes we compared (Additional file 2: Table SE10; Methods). Particularly, 861 of the *Pst* (93–210) and 955 of the *Psh* (93TX-2) proteins do not have any orthologs in the other 14 fungi or the NCBI database, and therefore are exclusively present in *Pst* (93–210) and *Psh* (93TX-2), respectively. Their absence in the other haplotigs due to polymorphisms between two nuclei [23] in a dikaryon was also confirmed (Methods). Indeed, 381 [out of 861 in *Pst* (93–210)] and 341 [out of 955 in *Psh* (93TX-2)] proteins had significant hits in the protein database, and all significant hits were within genus *Puccinia* [mainly the 2 K-41-Yr9 (PST-78) isolate] [20]. The absence of these genes in *Pst* (93–210) or *Psh* (93TX-2), but presented in other *Pst* isolates, indicated them actually isolate-specific. However, the majority of the isolate-specific genes, 56% (480 of the 861 genes) in *Pst* (93–210) and 64% (614 of the 955) in *Psh* (93TX-2), did not have significant hits in the protein database, suggesting that these non-hit genes could be forma specialis specific. Taking together, these isolate-specific genes are rapidly evolving after speciation within the *Puccinia* genus.

**Fig. 5:**
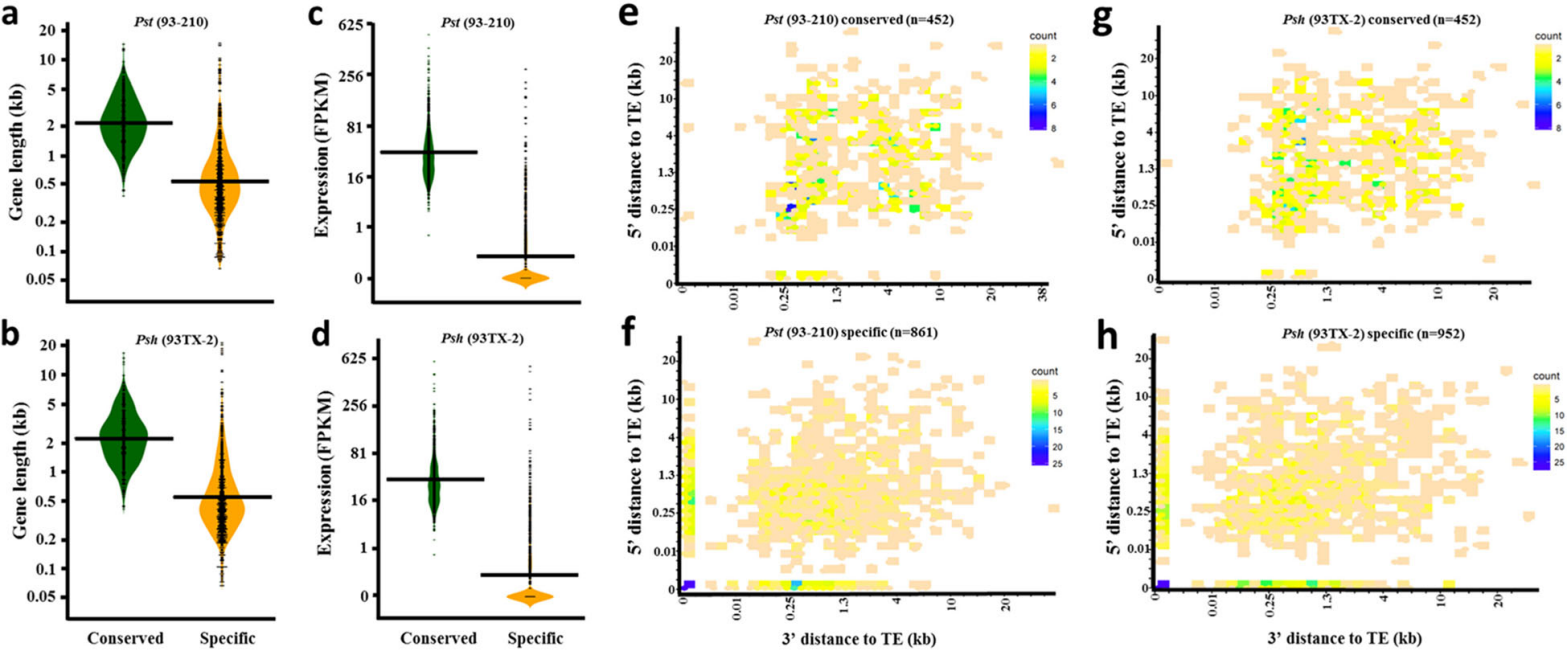
Distinct features of isolate-specific genes compared to conserved genes. **a**, **b** Gene length. **c**, **d** Gene expression level normalized in FPKM units. FPKM, fragments per kilobase of transcript per million read pairs. **e**-**h** Distances of genes to their closest transposable elements (TEs) at the 5′ and 3′ ends. A large number of isolate-specific genes are interrupted by TEs, and therefore denoted as 0 bp away to the closest TEs in **f** and **h**

**Fig. 6:**
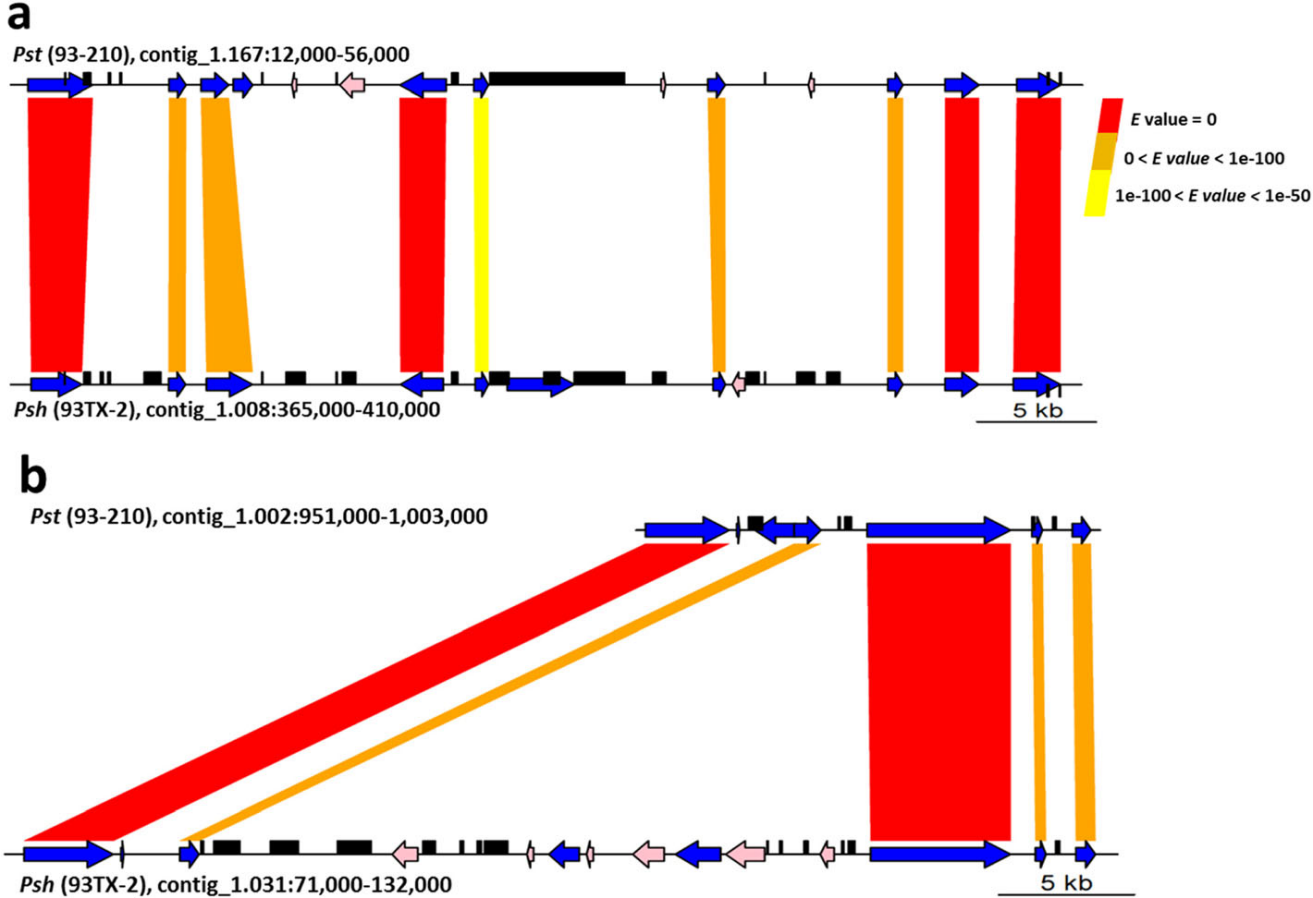
Genomic environment of isolate-specific genes. See the legend of Fig. 4. Pink arrows are isolate-specific genes. The ranges of E values for the homologies in the corresponding genomic regions between *Psh* and *Pst* for both Fig. 6a and b are given in Fig. 6a

The isolate-specific genes had a mean of 876 bp in length and were significantly shorter than the mean of conserved genes [*p* = 1.72e-59 for *Pst* (93–210) and 4.59e-56 for *Psh* (93TX-2); two-tailed student *t*-test] (Fig. 5a, b). The specific genes in both isolates had significantly fewer RNA sequencing reads than the conserved genes [*p* = 6.01e-48 for *Pst* (93–210) and 6.17e-40 for *Psh* (93TX-2), two-tailed student *t*-test] (Fig. 5c, d). Indeed, no transcripts were identified by the RNA-Seq analysis for 65% [557 out of 861 in *Pst* (93–210), and 621 out of 955 in *Psh* (93TX-2)] of the isolate-specific genes (FPKM = 0); whereas all of the conserved genes were expressed. We also investigated the distances of the isolate-specific genes to the closest TEs. In *Pst* (93–210), the mean distances of isolate-specific genes to the closest TEs were 1612 bp at the 5′ ends and 1665 bp at the 3′ ends, and these distances were significantly shorter (*p* = 2.57e-13 and 1.61e-15) than those of the conserved genes, 3323 and 3560 bp, respectively (Fig. 5e, f). Similarly, the significantly different distances between isolate-specific genes and conserved genes to the closest TEs were also observed in *Psh* (93TX-2) (Fig. 5g, h). Specifically, we noticed that 201 (out of 861) in *Pst* (93–210) and 219 (out of 955) genes in *Psh* (93TX-2) were interrupted by TEs at the 5′ and/or 3′ ends. Two examples of the relationship between isolate-specific genes and TEs are shown in Fig. 6a, b.

Given the significant low level of expression, we hypothesize that the rapidly evolving isolate-specific genes may be dispensable in biological functions. By searching the KEGG GENES database, 91.8% (790 out of 861) in *Pst* (93–210) and 90.7% (866 out of 955) in *Psh* (93Tx-2) of isolate-specific genes could not be assigned to any KEGG pathways (Additional file 2: Table SE11). In contrast, 80.5% (364 out of 452) of the highly conserved genes were successfully assigned to different KEGG pathways including basic biological processes such as genetic information processing (transcription, translation, replication and repair) and metabolic pathways (carbohydrate, amino acid, lipid and nucleotide metabolisms). More interestingly, most [66 out of 71 in *Pst* (93–210) and 80 out of 89 in *Psh* (93TX-2)] of the isolate-specific genes that could be assigned to KEGG pathways were accompanied by at least one KEGG orthologous gene, indicating that these genes are actually redundant genes in the pathway (Additional file 2: Table SE11). Moreover, we did not detect any particular GO terms that were significantly enriched in isolate-specific proteins comparing with the rest of the proteins in both *Pst* (93–210) and *Psh* (93TX-2). In summary, our data strongly supported that these rapidly evolving isolate-specific genes may be dispensable in essential functions.

Through reciprocal mapping of RNA-Seq reads, we identified 116 and 119 genes that were isolate-specifically expressed in *Pst* (93–210) and *Psh* (93TX-2), respectively, but silent in the other isolate (Additional file 1: Figure S7). These exclusively expressed genes were randomly dispersed throughout the genome. Among these genes, we observed slightly different numbers of SPs (22 versus 7) and carbohydrate active enzymes (5 versus 0) between *Pst* (93–210) and *Psh* (93TX-2). Also more exclusively expressed genes were annotated with GO terms in *Pst* (93–210) than *Psh* (93TX-2) (23 versus 13). The majority of the annotated GO terms were related to biological processes. Similar to isolate-specific genes, only 6.89% (8 out 116) and 3.36% (4 out of 119) were assigned to KEGG pathways, and all of them were redundant under the KEGG orthology. These results indicated that the isolate-specifically expressed genes were also dispensable and might have lost functions in the isolate in which these genes were silent.

### Differences in telomere repeats

In addition to the different functional genes, *Pst* (93–210) and *Psh* (93TX-2) are significantly different in the number and pattern of telomere repeats. The *Pst* (93–210) genome had two telomere repeat patterns (Table 5). The first repeat pattern was characterized by the presence of multiple copies (> 10) of (TTTTAGGG) followed by multiple copies (> 30) of (TTAGGG); while only (TTAGGG) is presented multiple times (> 40) in the second repeat pattern. *Pst* (93–210) had both patterns in eight contigs, whereas *Psh* (93TX-2) had only the second pattern in five contigs. All telomere repeats were located in the ends of these contigs, indicating the completeness of the assembled telomere regions.

**Table 5:** Telomere repeats and organization in isolates *Pst* (93–210) and *Psh* (93TX-2) genomes of *Puccinia striiformis*

Contig name	Contig size (bp)	Start of telomeric array	End of telomeric array	Size of telomeric array (bp)^a^	Position of telomere on contig	Number of telomeric repeats (5′-3′)
*Pst* (93–210)						
Contig1.035	512,194	511,887	512,194	307	end	(TTTTAGGG)_11_(TTAGGG)_38_
Contig1.074	318,575	318,319	318,575	257	end	(TTTTAGGG)_17_(TTAGGG)_21_
Contig1.124	232,724	232,418	232,724	307	end	(TTTTAGGG)_16_(TTAGGG)_32_
Contig1.178	156,450	156,143	156,450	307	end	(TTTTAGGG)_10_(TTAGGG)_40_
Contig1.186	147,797	147,519	147,797	279	end	(TTTTAGGG)_10_(TTAGGG)_35_
Contig1.196	140,041	139,736	140,041	306	end	(TTTTAGGG)_8_(TTAGGG)_40_
Contig1.227	113,221	112,944	113,221	268	end	(TTTTAGGG)_10_(TTAGGG)_33_
Contig1.423	31,619	31,319	31,619	301	end	(TTTTAGGG)_14_(TTAGGG)_32_
Contig1.026	542,491	542,248	542,491	244	end	(TTAGGG)_42_
Contig1.083	295,440	295,160	295,440	281	end	(TTAGGG)_47_
Contig1.087	289,332	289,039	289,332	294	end	(TTAGGG)_49_
Contig1.127	230,054	229,794	230,054	261	end	(TTAGGG)_46_
Contig1.151	197,251	197,012	197,251	240	end	(TTAGGG)_40_
Contig1.206	132,791	132,533	132,791	259	end	(TTAGGG)_43_
Contig1.212	128,166	127,952	128,166	215	end	(TTAGGG)_35_
Contig1.284	76,604	76,345	76,604	260	end	(TTAGGG)_43_
*Psh* (93TX-2)						
Contig1.118	212,623	212,376	212,623	248	end	(TTAGGG)_42_
Contig1.162	163,479	163,194	163,479	286	end	(TTAGGG) _47_
Contig1.169	159,050	158,860	159,050	191	end	(TTAGGG)_31_
Contig1.300	84,330	84,093	84,330	238	end	(TTAGGG)_40_
Contig1.402	53,760	53,511	53,750	240	end	(TTAGGG)_42_

^a^Size of telomeric array is the size in the assembly. Because there are miss-assembled deletions in the telomere regions, this size is not equal to the calculated size from the last column

## Discussion

In this study, we focused on deciphering the genomic basis underlying the host adaptation of filamentous plant pathogens at the forma specialis level. To this end, we generated genome references for two formae speciales in *P. striiformis*, namely *P. striiformis* f. sp. *tritici* using isolate *Pst* (93–210) that is highly adapted to wheat and *P. striiformis* f. sp. *hordei* using isolate *Psh* (93TX-2) that is highly adapted to barley (Fig. 1). Our genomic analyses showed that the host adaptation of formae speciales is a complex trait in *P. striiformis*, supported by the following two factors: i) no specific gene (or family) loss or gain could explain their adaptation, even though the two formae speciales were slightly different in specific protein family compositions (Table 2); and ii) a large number of both non-SP and SP genes were under diversifying selections, in contrast to *B. graminis* f. sp. *tritici* and *B. graminis* f. sp. *hordei* in which SP genes have much higher dN/dS ratios compared to non-SP genes [24]. However, we found the following genomic features associated with the host adaptations of *Pst* and *Psh*: i) the high genetic variations between two nuclei in a urediniospore provide raw genetic materials for genome evolution; ii) the rapidly evolving isolate-specific genes are dispensable and subject to functional loss; iii) gene loss may be adaptive during the host adaptation; and iv) transposable elements accelerate the evolution of host adaptation by involving in gene loss.

### High genomic variations within dikaryotic urediniospores

High heterozygosity as a function of genetic variation within dikaryotic urediniospores has been observed both in individual isolates and at the population level in *P. striiformis* [19, 22, 29, 30]. The origin of the heterozygosity remains unclear, e.g. whether via plasmogamy of two compatible but genetically distant haploid nuclei, spontaneous mutation or both. A large proportion of heterozygous genome regions and genes shared between *Pst* (93–210) and *Psh* (93TX-2) in this study (data not shown) indicates that both haplotypes and alleles had existed before the divergence of the two formae speciales. The spontaneous mutations might occur during and/or after the divergence. Regardless of the origin of the heterozygosity, our study clearly demonstrated that many of the genes were highly affected by mutations (Additional file 3: Tables S4 and S5). The impacts of the mutations might be two-sided. For instance, some mutations caused gene losses through interrupting start codons; while other mutations might create new genes through gaining stop codons. Through these ways, an isolate is able to obtain new gene functions by changing one allele and keeping the other allele functional. This can be supported by the recently identified *AvrSr50* gene in the wheat stem rust pathogen (*P. graminis* f. sp. *tritici*) in which the avirulence allele was functional while the insertion-disrupted virulence allele was not expressed [31]. This process might be particularly beneficial for *P. striiformis* in the geographic regions where only asexual stages exist [32–35]. This is consistent with our previous observation that *Pst* isolates with high homozygosity are less adaptive [29]. Besides mutations, Schwessinger et al. [23] recently observed another form of heterozygosity as one allele being totally lost for many genes in an Australian *Pst* isolate. Taking together, these evidences supported our hypothesis that high genomic variations within dikaryotic urediniospores provide raw genetic materials for the rapid genome evolution of *P. striiformis*.

### Isolate-specific genes subject to gene loss

From protein family analysis, we observed a large number of protein-coding genes unable to assign to any families, suggesting that they are species- (or forma specialis-) specific among the 16 fungal genomes compared. The number of specific genes in a genome ranged from 3.2% (210 genes in *B. graminis* f. sp. *hordei*) to 17.8% (2845 in *P. graminis* f. sp. *tritici*). It should be noted that the number of specific genes detected largely depends on the reference genome sets selected, meaning that the more closely related samples used, the less number of specific genes could be detected. This explains the relatively lower percentage of specific genes in *B. graminis* f. sp. *hordei* and *B. graminis* f. sp. *tritici*, and in *Pst* (93–210) and *Psh* (93TX-2) as they are different formae speciales under a species in each pair. Even so, we still were able to detect a relatively large number of specific proteins at the forma specialis level for these fungi. Particularly, 872 and 967 proteins of *Pst* (93–210) and *Psh* (93TX-2) did not have any homologous in the remaining 14 fungi genomes compared. Manual examination of these proteins in the haplotigs of assembled *Pst* (93–210) and *Psh* (93TX-2) genomes detected 11 and 12 proteins (tblastn, e-value <1e-100, identity > 90), respectively. Therefore, we concluded that 861 (=872–11) and 955 (=967–12) proteins are exclusively present in *Pst* (93–210) and *Psh* (93TX-2), respectively.

We found that the FPKM values of 128 genes in *Pst* (93–210) was > 1.0 when the RNA-Seq reads of *Pst* (93–210) were mapped, while the expression level was 0 when the RNA-Seq reads of *Psh* (93TX-2) were mapped. This indicated that these 128 genes were expressed during the *Pst* (93–210) infection, but their respective homologous genes, if they have, did not express at all in *Psh* (93TX-2). We also noticed that among these 128 genes, 12 genes were actually *Pst* (93–210)-specific genes and therefore do not have homologous genes in *Psh* (93TX-2). As the 12 genes have potential for developing molecular markers for monitoring the pathogen populations and studying their functions, they are present in Additional file 2: Table SE12. However, these 12 genes were excluded from the analysis for isolate-specific expression. The remaining 116 genes were considered as exclusively expressed genes in *Pst* (93–210), and similarly, we identified 119 genes that were exclusively expressed in *Psh* (93TX-2) (Additional file 2: Table SE13). The exclusively expressed genes in the *Pst* or *Psh* isolates may contribute to their host adaptation.

Genomes of many filamentous plant pathogens harbor a large number of lineage-specific genes (Additional file 2: Table SE10; [36]), called orphan genes that lack homologues in other lineages [37]. In the present study, we identified the following three groups of isolate-specific genes in either *Pst* (93–210) or *Psh* (93TX-2). The first group includes the genes that are present in the other fungi and present in either *Pst* (93–210) or *Psh* (93TX-2) but not both. These genes have been involved in gene-loss events since the divergence of *Pst* and *Psh* (Fig. 4a). The second group contains the isolate-specific genes that are present in either *Pst* (93–210) or *Psh* (93TX-2), but absent in all other fungi. These genes have been involved in gene-gaining events since the divergence of *Pst* and *Psh*. These isolate-specific genes are distinct from the conserved genes in that they are significantly shorter in length, less expressed and closer to TEs (Fig. 5), suggesting that they are ‘younger’ genes and fast-evolving [37]. The third group includes the genes that are present in both *Pst* (93–210) and *Psh* (93TX-2), but exclusively expressed in either *Pst* (93–210) or *Psh* (93TX-2). In contrast to many other comparative analyses at the species or genus level with emphasis specifically on secreted effector genes [38], the SP genes identified in the present study are not enriched as specific genes for *Pst* (93–210) or *Psh* (93TX-2) (Table 4). These results indicate that the effector genes are at least not the only determinant of host specificity in these formae speciales of *P. striiformis*, and that the role of non-effector genes might be underestimated. This finding is different from the previous report of forma-specialis specific effectors in *Fusarium oxysporum* [39]. Similar to virulence-related genes, the specific genes in *Pst* (93–210) or *Psh* (93TX-2) are also located in plastic genomic environments enriched by TEs (Figs. 4, 5e-h, 6). The emergence of specific genes was not due to horizontal gene transfer (see Methods), and less possible by de novo evolution of genes from non-coding sequences given the relative short divergence time between *Pst* (93–210) and *Psh* (93TX-2). Instead, we speculate that the presence of specific genes in one isolate (or forma specialis) resulted from the loss of the homologues in the other isolate (or forma specialis) by replacements of TEs or loss of genomic fragments (Figs. 4b, c, 6). While functions of lineage-specific genes remain unknown, we postulate that, particularly in *P. striiformis*, the specific genes are dispensable and subject to gene loss. This hypothesis is supported by their lower levels or absence of expression (Fig. 5c, d, Additional file 1: Figure S7).

### Gene loss as putative genomic basis for host adaptation

We summarize that gene loss is an underestimated genetic mechanism during host adaptation in filamentous plant pathogens. As we have shown above, the specific genes in *Pst* (93–210) and *Psh* (93TX-2) are the direct consequence of gene loss. Remarkably, pervasive gene-loss events after speciation were observed in all fungi we examined in the present study (Fig. 4a). This is slightly different from the previous prediction [40] since closely related plant pathogen species (and formae speciales) were selected in our study and therefore possible homologues could be distinguished at a higher power [37]. In fact, gene losses have been reported in many filamentous plant pathogens to provide evolutionary adaptive responses to changes in host and environmental conditions, and even associated with pathogen lifestyles [40]. For instance, reductions of genes encoding carbohydrate active enzymes, secondary metabolites and enzymes in nitrogen and sulfur assimilation pathways enable biotrophs adapted to plant hosts by avoiding recognition by plant defense systems [25, 41]. Moreover, the loss of an *Avr* gene is an effective way of host adaptation for many plant pathogens in agro-systems in which the corresponding *R* genes change rapidly in both space and time, e.g. *AvrLm1* in *Leptosphaeria maculans* [42], *AVR-Pita* in *Magnaporthe oryzae* [43] and *NIP1* in *Rhynchosporium secalis* [44]. Particularly in the recently identified *AvrSr35* in *P. graminis* f. sp. *tritici*, the loss of *AvrSr35* resulted from insertion of mobile element enabled avirulent isolates virulent to the *Sr35* resistance gene [45].

We speculate that losses of some of the isolate-specific genes may be adaptive for *Pst* (93–210) and *Psh* (93TX-2) to infect their respective hosts. We provide the following reasons. Hyposensitive-reaction (HR) was observed when barley cultivar Steptoe was inoculated with most *Pst* isolates (Fig. 1b), suggesting that most *Pst* isolates have *Avr_barley* genes corresponding to the resistance genes in Steptoe [46]. Such *Avr_barley* genes in *Pst* must be lost or mutated in *Psh*, since no HR was observed when Steptoe was inoculated with all *Psh* isolates (Fig. 1c) [47]. Similarly, most *Psh* isolates should have *Avr_wheat* genes corresponding to the resistance genes in Nugaines (Fig. 1d), while such *Avr_wheat* genes in *Psh* must be lost or mutated in *Pst* since Nugaines is susceptible to all *Pst* isolates (Fig. 1a) [47–49]. In summary, *Pst* has lost the *Avr_wheat* genes presented in *Psh*, and therefore highly adapted to wheat; while *Psh* has lost the *Avr_barley* genes presented in *Pst*, and therefore highly adapted to barley*.* This is consistent with the isolate-specific genes identified in *Pst* (93–210) and *Psh* (93TX-2). Further studies are needed to characterize such genes from the hundreds of genes specific to the isolates identified in the present study.

Mutations in protein-coding DNA sequences are classified as either synonymous when they do not change the encoded amino acids, or nonsynonymous when they can change the amino acids. Since these two substitutions are under different selective pressures, the comparison of synonymous and nonsynonymous substitution rates (dN and dS, respectively) between paired protein-coding DNA sequences can indicate the action of natural selection on the proteins. We found that a large number of both SP- and non-SP-coding genes were positively selected during the *Pst* and *Psh* divergence and that genes under purifying selections are enriched for specific families. More SP-coding genes were under positive selection than those under purifying selection. The putative SP genes under positive selection are involved in diverse pathways. These results suggest positive selection for some of the SP genes, which may have contributed to the adaptations to different cereal crops.

### Differences in telomeres

The numbers and patterns of telomere repeats in the *Pst* (93–210) and *Psh* (93TX-2) suggest different chromosomal structures between these two isolates or formae speciales. To our knowledge, this is the first report of telomere repeats in rust fungi. The repeat TTAGGG is the same as the telomeric repeat reported in vertebrates and some filamentous fungi, while TTTTAGGG is the same as the repeat in green algae (*Chlamydomonas* spp.) [50, 51]. Eight contigs containing telomere repeats of (TTTTAGGG)_n_(TTAGGG)_n_ and eight contigs containing telomere repeats of (TTAGGG)_n_ were found in *Pst* (93–210), whereas only five contigs containing (TTAGGG)_n_ were found in *Psh* (93Tx-2). It is not clear whether the different numbers of contigs containing telomeric repeats indicate different numbers of chromosomes in these isolates. Based on the base number of 6 chromosomes and the dikaryotic number of 12 chromosomes in dikaryotic urediniospores of *P. striiformis* previously determined by cytological and linkage analyses [52, 53], the five pairs of telomeric regions observed in *Psh* (93TX-2) indicate that the six chromosomes in one nucleus have identical numbers of the repeat in both telomeric regions of each chromosome and two of the chromosomes have identical numbers of the repeat. In contrast, the eight pairs of different repeats observed in *Pst* (93–210) indicate that each of the six chromosomes have different repeats in the telomeric regions [one containing (TTAGGG)_n_ and one containing (TTTTAGGG)_n_(TTAGGG)_n,_ and the two nuclei in a urediniospore have different sets of chromosomes. The differences in telomeric regions reflect chromosomal changes during the divergence of these two isolates and further work needs to be done to determine if the different numbers and patterns of telomeric repeats are conserved within each of the formae speciales. As the number of telomere repeats is associated to aging, disease and environment in humans and other vertebrates [54], the different telomeric repeats resulting from evolution may contribute to host and/or environment adaptations.

### Evolution of host adaptation of *P. striiformis* to wheat and barley

**Fig. 7:**
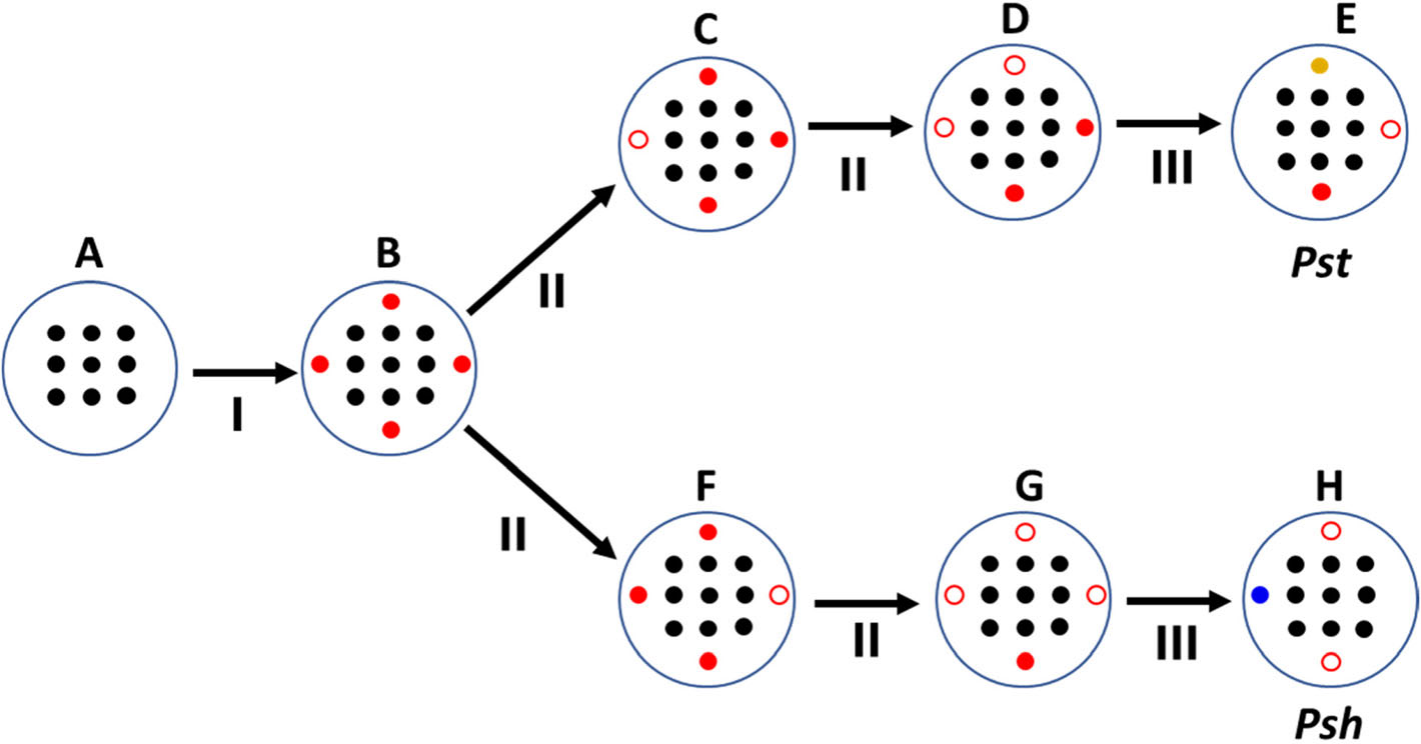
A simplified evolutionary scenario for the host adaptation of *Puccinia striiformis* f. sp. *tritici* (*Pst*) and *P. striiformis* f. sp. *hordei* (*Psh*). **a**-**h**: genomes; **a**, the most recent common ancestor of *Puccinia* spp.; **b**, the most recent common ancestor of *P. striiformis*; **c**-**d** and **f**-**g**, intermediate ancestor of *Pst* (**e**), and *Psh* (**h**), respectively. **I**-**III**: evolutionary processes; **I**, gene family gains and expansions during speciation; **II**, gene losses; **III**, gene losses, and/or gains, host adaptation. Black circles, conserved genes; colored circles, rapid evolving genes; solid circles, functional genes; and empty circles, loss-of-function genes

Based on our genomics analyses of *Pst* and *Psh* genomes, we propose a simplified evolutionary scenario for the host adaptation of these two formae speciales, as shown in Fig. 7. The most recent common ancestor of *Puccinia* spp. experienced extensive gene-family gains and expansions (Fig. 4a) [36], accompanied by host jumps and shifts [55]. Then the divergence of formae speciales occurred several million years after the divergence of wheat and barley at 11.6 Mya (Fig. 3a) [27]. The divergence of *Pst* and *Psh* were concurrent with mutations (Table 3; Additional file 3: Tables S4 and S5), sexual recombinations [22], somatic recombinations [56] and other evolutionary forces [57], leading to extensive gene losses and occasionally gene gains (Fig. 4a), which provide the genomic basis for host adaptation at the formae speciales level. These evolutionary processes are accompanied and accelerated by massive TE activities as reported for other fungi [4]. We further postulate that the host adaptation is an ongoing evolutionary process and the formae speciales in *P. striiformis* may have incomplete reproductive isolation in agro-ecosystems.

## Conclusions

In this study, we used comparative genomics and analyzed genomes of two isolates, *Pst* (93–210) and *Psh* (93TX-2) that are highly adapted to wheat and barley, respectively, to represent two economically important formae speciales of *P. striiformis*. Based on our analyses, we conclude that i) host adaptation at the forma specialis level is a complex trait, involving not only virulence-related genes but also other genes; ii) gene loss, along with other evolutionary processes, provides the genomic basis for host adaptation in *P. striiformis*; and iii) the evolution of host adaptation is accompanied and driven by TE activities. In addition, changes in telomeric regions may contribute to host adaptation. Our results suggest that gene loss is more important than expected in the evolution of host adaptation in filamentous plant pathogens, at least at the forma specialis level.

## Methods

### Pathogenicity test

We selected two isolates, *Pst* (93–210) and *Psh* (93TX-2) that were collected from Montana and Texas, respectively in 1993 in the US, to represent wheat and barley stripe rust pathogens, respectively [13]. We first determined their host adaptation using pathogenicity tests. For both *Pst* (93–210) and *Psh* (93TX-2) isolates, fresh urediniospores were used to inoculate both Nugaines and Steptoe. Nugaines is a wheat cultivar and susceptible to all *Pst* isolates collected in the US, whereas Steptoe is a barley cultivar and susceptible to all *Psh* isolates collected in the US [13, 47–49, 53]. We followed an inoculation procedure described previously [58]. Virulence phenotypes were recorded, and photos were taken 15 days after inoculation. The two isolates were also tested on the 18 wheat *Yr* single-gene lines used to differentiate *Pst* races [58] and 12 barley cultivars used to differentiate *Psh* races [47].

### Genome sequencing and read preparation

Genomic DNA was extracted from urediniospores using the CTAB method [59]. A DNA library with fragment sizes of approximately 150 bp was prepared and sequenced using the Illumina HiSeq PE 150 technology. Meanwhile, a PacBio RS II library was constructed for both isolates and sequenced using P4-C2 chemistry with 16 SMRT cells, generating 5.55 and 6.27 Gb reads for *Pst* (93–210) and *Psh* (93TX-2), respectively. Trimmomatic (version 0.36) [60], Lighter [61] and Proovread v2.14.0 [62] were used to correct the potential sequencing errors in the Illumina and PacBio long reads. Genome assembly and annotation were outlined in Additional file 1: Figure S8. More detailed steps for assembly, polishing, and annotation can be found in our related paper [63].

One additional *Psh* isolate (04–051) was sequenced using the Ion Proton technology following the previously described method [64]. This isolate was collected from Oregon, Washington in 2004 and was virulent on barley differentials Topper, Heils Franken, Hiproly, Abed Binder 12, Trumpf, I 5 and Bancroft but avirulent on the remaining barley differentials (Emir, Astrix, Varunda, Mazurka and Bigo) and all wheat differentials for identifying *Pst* races. The sequences of this isolate was used to determine whether the genes specifically present or absent in *Psh* (93TX-2) were also the same in this isolate.

### *K-*mer analysis and genome size estimation

Program Jellyfish version 2.2.6 [65] was used to analyze the distribution of *K*-mer and to estimate the genome size using only filtered and corrected Illumina reads. According to the Lander-Waterman theory [66], the genome size can be estimated using the total number of *K-*mers divided by the peak value in the *K-*mer distribution. We found that both *Pst* and *Psh* isolates were heterozygous since both commonly had two peaks in the *K-*mer distribution (Additional file 1: Figure S1). Then the genome size was calculated separately for regions with and without heterozygosity. With the peak at the expected *K-*mer depth, the calculated genome size, which equals the total *K-*mer/expected *K-*mer depth, was estimated for each region.

Two peaks of 17-mer were observed at the depth of 19 and 33 for isolate *Pst* (93–210), representing two distinct heterozygous and homozygous genomic regions, respectively. The total estimated genome size was 89,743,503 bp, of which 20,535,944 bp and 69,207,559 bp were estimated to be in the heterozygous and homozygous regions, respectively. Similarly, two peaks at 15 and 26 were observed for isolate *Psh* (93TX-2). The estimated genome size was 89,709,453 bp, of which 18,880,342 bp and 70,829,111 bp were estimated to be in the heterozygous and homozygous regions, respectively.

### Analysis of telomeres

The gapless haploid genomes in this study enabled us to accurately survey the telomere regions that might be often unassembled in previously available cereal rust genomes. Telomere repeats were identified by manually searching the ends of contigs for short, high-copy-number (> 30) repeats.

### Transcriptome sequencing

RNA sequencing was conducted for both *Pst* (93–210) and *Psh* (93TX-2). Total RNA was extracted from fresh urediniospores, germinated urediniospores and infected wheat leaves 2 and 7 days after inoculation. Fresh urediniospores, germinated urediniospores and infected leaves were grounded separately in a mortar in liquid nitrogen. Total RNA was extracted from frozen powder using the Trizol reagent (Thermo Fisher Scientific, MA) following the manufacturer’s instruction. RNA quality was checked using the Agilent®2100 Bioanalyzer® (Sgilent Technologies, UK). The RNA samples from different stages of each isolate were mixed into one sample at the 1:1:1:1 ratio for RNA sequencing. The preparation of RNA-Seq library and Illumina HiSeq PE150 sequencing were performed at the Novogen Corporation Inc. (Sacramento, CA). Raw reads of RNA-Seq were checked and trimmed using the same methods as described above. In total, 19,802,677 and 25,432,126 paired-end reads of *Pst* and *Psh* were obtained and used for subsequent analyses. The filtered RNA-Seq reads were used for both genome annotation and gene expression estimation.

### SNP and InDel analyses

A previously proposed framework for discovering variation was followed [67]. To identify intra-isolate SNPs and InDels, the high quality Illumina reads of each isolate were mapped to its respective assembled genome as a reference, and high quality SNPs and InDels were called from the alignments. The reference genome was indexed using the Burrows-Wheeler Alignment (BWA) Tool version 0.7.15 [68]. High quality paired-end Illumina reads were mapped to the reference using the mem algorithm with the default parameters except -r 1.0. The SAM formatted alignment was converted to the BAM format after removing the duplicates using SAMtools version 1.2 [69]. The BAM files were cleaned, validated and sorted using the Picard Tools version 1.129 (http://picard.sourceforge.net/), and then indexed using SAMtools. To reduce the number of potentially mismatching Indels in the alignment, the local realignment was conducted following two steps using the Genome Analysis Toolkit (GATK) version 3.3 [67]. First, the interval targets for local realignment were defined using GATK’s RealignerTargetCreator with the default parameters. Second, GATK’s IndelRealigner was used to perform indel realignment of reads around defined targets from the previous step.

To improve the accuracy of base qualities for subsequent variant calling, we performed a Base Quality Score Recalibration step to detect systematic errors made during the sequencing when estimating the quality score of each base call. Firstly, we performed an initial round of variant calling using GATK HaplotypeCaller. SNPs and InDels of high confidence were filtered based on the Quality value in vcf using vcftools (version 0.1.13) with parameters set as --min-alleles 2 --max-alleles 2 --minQ 1000 (meaning that only biallelic SNPs and InDels with a minimum Quality of 1000 were kept). Then the filtered high confidence SNPs and InDels were used as known variants to model systemic sequencing errors empirically, and the base qualities were adjusted using GATK BaseRecalibrator. Lastly, the recalibrated BAM was generated using GATK PrintReads. The second round of variant calling was performed using GATK HaplotypeCaller. To finalize, the high quality SNPs and InDels with minimum calling quality of 100 were selected. Furthermore, we only kept SNPs and InDels within coverages 20-120X to reduced false positives in repetitive regions where Illumina reads from multiple different copies of the repeat were mapped to a single locus. These were done using vcftools with the parameters set as --min-meanDP 20 --max-meanDP 120 –minQ 100.

The variations (SNPs and InDels) were annotated and their effects were predicted using software SnpEff version 4.3r [70]. Firstly, the databases of *Pst* and *Psh* were built locally using annotations in the GTF format generated from the genome annotation section (described above). The SnpEff databases were subsequently used to annotate intra- and inter-isolate SNPs and InDels with putative functional effects according to categories defined in the SnpEff v4.3r manual. Other parameters were set as default, e.g. the upstream and downstream interval sizes of 5000 bp. Predicted effects are given in Additional file 3: Table S4. As defined by SnpEff, some of the high impacts include exon_loss, frameshift, splice_site_acceptor, splice_site_donor, start_loss, stop_lost and stop_gained. It should be noted that SnpEff allows variations to be included in multiple categories, so the total number of effects does not exactly match the total number of variants in Additional file 3: Table S4.

### Synteny analysis

Whole genome comparison between *Pst* (93–210) and *Psh* (93TX-2) was conducted to evaluate gene synteny using the MUMmer 3.23 package [71]. Specifically, the alignments of nucleotide contigs were generated from NUCmer using the option --maxmatch set to include all match sequences regardless of uniqueness and the defaults for other parameters. The delta encoded alignments were filtered using delta-filter by setting -i 90 -l 1000 -r -q -o 10 to keep only the alignments with identities above 90% and length above 1000 bp. For visualization, plots were generated using mummerplot and Gnuplot, visualized using program circos version 0.69–5 [72]. The total size of syntenic regions was calculated by adding length of all alignments, and the overall identity was calculated by averaging the identities of all aligned blocks. Also compared were *Pst* (93–210) versus *Pst* (104E 137A-) and *Psh* (93TX-2) versus *Pst* (104E 137A-) [23].

### Ortholog detection and analysis

Protein orthologous groups (orthogroups) were inferred using OrthoFinder version 1.1.8 [73] with the default parameters. Protein sequences of 16 plant fungal pathogens including 4 Ascomycetes and 12 Basidiomycetes were retrieved from the NCBI or JGI database (Additional file 2: Table SE9). To infer orthogroups, an all-vs-all BLAST search was firstly performed, and then the resulting e-values were used for clustering using the MCL program.

The isolate-specific genes, 861 in *Pst* (93–210) and 955 in *Psh* (93TX-2), were searched against the NCBI non-redundant protein database (updated on March 6, 2017; downloaded September 25, 2017) using blastp v2.4.0 to identify their potential origin, e.g. via horizontal gene transfer (HGT). To identify their genomic features, we compared the isolate-specific genes in *Pst* (93–210) and *Psh* (93TX-2) with 452 single-copy protein-coding genes identified from 16 fungi using OrthoFinder. The reason behind this is that the isolate-specific genes represent a group of rapidly evolving genes in these isolates, whereas the 452 single-copy protein-coding genes are present in all 16 fungi and therefore are highly conserved. We first calculated the lengths of isolate-specific genes in *Pst* (93–210) and *Psh* (93TX-2). Secondly, we mapped our RNA-Seq reads to the respective reference genomes to estimate the gene expression level. Then the gene expression level was quantified from the gene abundance file generated using program StringTie v1.3.3b [74] and normalized in the number of fragments per kilobase of transcript per million read pairs (FPKM). Thirdly, we calculated the genomic distances of isolate-specific genes to the closest transposable elements (TEs) at both 5′ and 3′ ends, and compared the values to those of the conserved genes. These features were visualized using the ‘beanplot’ [75] (Fig. 5a-d) and ‘ggplot2’ [76] (Fig. 5e-h) packages in program R.

To test the hypothesis that the rapidly evolving isolate-specific genes may be subject to loss-of-function, the isolate-specific genes were searched against the nonredundant set of KEGG (Kyoto Encyclopedia of Genes and Genomes) genes using software GhostKOALA version 2.0 [77].

Statistical analysis of the GO-term enrichment was conducted using program Fatigo in Blbelomics 5 [78]. Specifically, we compared the GO terms: 1) of all proteins of *Pst* (93–210) and *Psh* (93TX-2); 2) of proteins involved in loss events with the rest of the proteins in *Pst* (93–210) and *Psh* (93TX-2); and 3) of isolate-specific proteins with the rest of the proteins in *Pst* (93–210) and *Psh* (93TX-2). No significant GO term enrichment was detected in all comparisons.

### Divergence time and mutation rate

The proteins from 452 single-copy orthogroups were used to infer the phylogenomic relationship and divergence time of *Pst* and *Psh*. Peptide alignment was conducted using CLUSTAL Omega version 1.2.4 [79]. Only sites without gaps in the alignment and alignments with at least 50 amino acids in length were kept in the subsequent analyses, resulting in 158,428 amino acid sites and 439 proteins. Phylogenomics inference was conducted using the Bayesian-based method in program MrBayes v3.2.6 [80], following the procedure described previously [64]. A partitioned analysis was set up for MrBayes so that different models could be estimated and applied for each protein. For each protein, the evolutionary model was selected based on ProtTest 3.4.2 [81]. The divergence times of *Pst* and *Psh* and of other fungal plant pathogens used in this study were estimated using a semiparametric Penalized Likelihood (PL) method with the Truncated-Newton (TN) algorithm in r8s version 1.81 [82]. Cross-validation was used to explore the fidelity of the method to explain the branch length variation. To do this, we tried to optimize a range of smoothing parameters, at increments of 1.0, from − 8 to 2 on a log_10_ scale. For our dataset, the smoothing value of 1e-05 gave the lowest cross-validation score, and therefore was used to obtain the optimal estimates of the divergence time and rate. To do such estimation, at least one calibration time is needed. We calibrated the times on the tree based on the divergence between Ascomycota and Basidiomycota at the minimum age of 452 million years that was previously estimated when a fossil of *Paleopyrenomycites* was considered to be in Ascomycota [83]. Another reason we used this divergence time was that this calibration time generated very close divergence for *Blumeria graminis* f. sp. *tritici* and *B. graminis* f. sp. *hordei* compared to a previous estimation on the basis of substitutions in synonymous sites [9.22 myrs versus 6.3 (±1.1) myrs] [24]. To further validate our estimated divergence time between *Pst* and *Psh*, we calculated the substitution rate between these two genomes. We selected 5611 pairs of single-copy protein homologs of the *Pst* and *Psh* isolates (see the dN/dS analysis section) and identified 1,307,578 bp substitutions from a total length of 8,025,786 bp for these genes. Based on these data, we calculated the mutation rate as 2.0 × 10^− 8^ per base per year, which is within a previous reported *Pst* mutation rate range of 1.7 × 10^− 7^ to 8.6 × 10^− 8^ per base pair per generation based on 17,000 AFLP fragments [84].

### dN/dS analysis

The method of Yang and Nielsen [85] was implemented in program yn00 of the PAML package version 4.9e [86], and the transition/transversion rate bias and codon usage bias were counted for estimating the dN/dS ratios, following the previously described method [24]. Firstly, the 5611 pairs of single-copy protein homologs of *Pst* and *Psh* were determined using OrthoFinder and aligned using CLUSTAL Omega. Then the protein alignment was used as an anchor to generate the corresponding CDS alignment to assure the corresponding bases were aligned, which is an essential requirement for dN/dS analyses. This step was achieved using program PAL2NAL [87], which converts protein alignment and the corresponding DNA sequences into a codon-based DNA alignment. The outputs in the paml format were concatenated and subjected to the yn00 analysis for dN/dS estimation. The dN/dS ratios were assessed separately for the SP-coding genes and non-SP-coding genes to test if the two groups had different characteristics of natural selection. It should be noted that it is impossible to calculate dN/dS when there are nonsynonymous substitutions but no synonymous substitutions in a gene sequence (dN ‡ 0, while dS = 0). In these situations, we arbitrarily designated dN/dS = 100, and plotted as > 3 in Fig. 3b.

### Evolution analysis of protein-coding gene families

Protein-coding gene families of 16 plant fungal pathogens were assigned using OrthoFinder as described above. The evolution of protein family loss and gain was analyzed following the method described previously [41]. First, the presence and absence information of each family in each species was prepared manually and stored in the Phlylip format. Then the DOLLOP program from the PHYLIP version 3.696 [88] was used to reconstruct a phylogenic tree using the Dollo parsimony method, and also to estimate the protein family loss and gain events for each species and for internal nodes of the phylogenetic tree.

### Identification of isolate-specifically expressed genes

To identify genes exclusively expressed in either *Pst* (93–210) or *Psh* (93TX-2), the filtered RNA-Seq reads generated from both isolates were mapped to *Pst* and *Psh* separately using program HISAT2 v2.1.0. The alignments were converted and sorted in the BAM format. Then gene expression level was quantified from the gene abundance file generated using program StringTie v1.3.3b [74]. A gene was considered as form-exclusively expressed when the normalized expression level in FPKM was > 1.0 using the RNA-Seq reads from *Pst* or *Psh* but the FPKM was 0 using reads of the other isolate.

### Analysis of specific protein-coding gene families

Putative SPs were predicted following a previously recommended pipeline [89, 90]. Briefly, the presence and location of signal peptide cleavage sites were predicated using SignalP version 3.0. The presence of secretory pathway signal peptide was confirmed using TargetP version 1.1. The proteins with predicted chloroplast transit peptides and mitochondrial targeting peptides were removed. The presence of transmembrane helices was predicted using TMHMM version 2.0 (http://www.cbs.dtu.dk/services/TMHMM/). Finally, the proteins with the presence of signal peptides and absence of neither chloroplast and mitochondrial targets transmembrane helices nor transmembrane helices were filtered for subsequent analyses.

To test whether specific carbohydrate-active enzymes were associated with host adaptation, we performed CAZyme annotation for two wheat-hosted fungi (*Pst* and *Bgt*) and two barley-hosted fungi (*Pgh* and *Bgh*). Predicted proteins of four fungi were searched against entries in the CAZy database (http://www.cazy.org) using the dbCAN Web server, an automated carbohydrate-active enzyme annotation pipeline based on the CAZyme family-specific domains [91].

Secondary metabolite (SM) anchor genes were predicted using program SMIPS [92] based on protein domain annotations using InterProScan as described above.

As membrane transporters, which have functions to transfer nutrients, metabolism products, signaling molecules and many other cellular constituents from source to sink for cellular uptake, are of particular importance for obligate biotrophic fungi, we searched membrane transporter genes. To do this, TCDB (Transporter Classification Database) sequences [93] in the fasta format were retrieved from http://www.tcdb.org/download.php (October 16th, 2017). The TCDB sequences were searched using Blastp (percentage identity > 30%; e-value <1e-50) to predict transporters in *Pst* (93–210), *Psh* (93TX-2), *Bgt* and *Bgh*.

Transcription factors were predicted based on the assignment of proteins to specific DNA-binding domain families using the Pfam libraries. The domain assignments were performed on complete genome proteins using InterProScan. Transcription factor families were classified as described in the transcription factor prediction database (http://www.transcriptionfactor.org).

Peptidases (also termed proteases, proteinases and proteolytic enzymes) and inhibitors were predicted in the four fungal (*Pst*, *Psh*, *Bgt* and *Bgh*) genomes using the *MEROPS* database (release 12.0) [94] which uses a hierarchical, structure-based classification of the peptidases. Proteins of the four fungi were searched against the *MEROPS* database, which was retrieved from ftp://ftp.ebi.ac.uk/pub/databases/merops/current_release/protease.lib (October 24th, 2017). The peptidase or inhibitor family of the best hit (percentage identity > 30%; e-value <1e-50) from the blastp results was assigned to the query protein.

### Coverage analysis and detection of genomic region deletions

Two *Pst* isolates (93–210 and 2K-41-Yr9) and two *Psh* isolates (93TX-2 and 04–051) were used to detect forma specialis-specific regions. The genomic sequence of 2K-41-Yr9 (PST-78) was from http://genome.jgi.doe.gov/pages/dynamicOrganismDownload.jsf?organism=Pucst_PST78_1 (Additional file 2: Table SE9), and the genomic sequence of *Psh* 04–51 was obtained in this study as mentioned above. We defined the genomic regions as *Pst-*specific regions when: 1) these regions were covered by mapping sequence reads of *Pst* isolates to the *Pst* reference genome; and 2) these regions were not covered by mapping sequence reads of *Psh* isolates to the *Pst* reference genome. Similarly, we also defined *Psh*-specific regions. The sequence reads of all four isolates were mapped to the *Pst* (93–210) and *Psh* (93TX-2) references using the method described above. We calculated the mapping coverage based on the recalibrated alignments in the BAM format using the bedcov module in SAMtools with a window size of 500 bp. The region was considered as deleted in the mapping isolate when the mapping coverage was 0. Selected deletion regions were visualized using Circos (Additional file 1: Figure S4).

## Additional files

Additional file 1: **Figure S1.** The 17-mer depth distributions of the Illumina reads used to estimate the genome sizes of *Pst* (93–210) and *Psh* (93TX-2) isolates of *Puccinia striiformis* used in this study. **Figure S2.** Comparisons of functional annotations between wheat-hosted and barley-hosted stripe rust and powdery mildew fungi. **Figure S3.** Collinearity analysis of syntenic blocks among three *Puccinia striiformis* isolates. **Figure S4.** Examples of missing genomic regions by reciprocal mapping of Illumina sequence reads. **Figure S5.** An overview of a large syntenic region between isolates *Ps* (93–210) and *Psh* (93TX-2) of *Puccinia striiformis*. 4. **Figure S6.** An overview of the mitochondrial (mt) genomes of isolates *Pst* (93–210) and *Psh* (93Tx-2) representing *Puccinia striiformis* f. sp. *tritici* (*Pst*) and *P. striiformis* f. sp. *hordei* (*Psh*), respectively. **Figure S7.** IGV screenshot showing examples of exclusively expressed genes revealed by reciprocal mapping of Illumina RNA-Seq reads. **Figure S8.** Genome assembly pipeline used for genome assembly in this study. See details in Methods. (DOCX 4535 kb)

Additional file 2: **Table SE1.** Summary of transposable elements in *Puccinia striiformis* f. sp. *tritici* and *P. striiformis* f. sp. *hordei* genomes. **Table SE2.** Comparison of the annotated CAZyme genes among *Puccinia striiformis* f. sp. *tritici* (*Pst*), *P. striiformis* f. sp. *hordei* (*Psh*), *Blumeria graminis* f. sp. *tritici* (*Bgt*) and *B. graminis* f. sp. *hordei* (*Bgh*). **Table SE3.** Mating-related genes in *Puccinia striiformis* f. sp. *tritici* (*Pst*), *P. striiformis* f. sp. *hordei* (Psh), *Blumeria graminis* f. sp. *tritici* (*Bgt*) and *B. graminis* f. sp. *hordei* (*Bgh*). **Table SE4.** Comparison of the annotated peptidases genes among *Puccinia striiformis* f. sp. *tritici* (*Pst*), *P. striiformis* f. sp. *hordei* (*Psh*), *Blumeria graminis* f. sp. *tritici* (*Bgt*) and *B. graminis* f. sp. *hordei* (*Bgh*). **Table SE5.** Comparison of the annotated genes in isolate *Pst* (93–210) (**Table SE5a**) and *Psh* (93TX-2) (**Table SE5b**) using the Pathogen-Host-Interaction database. **Table SE6.** Summary of secondary-metabolite gene classes in 17 fungal plant pathogens. **Table SE7.** Comparison of the annotated transcription factor genes among nine fungal species or formae speciales. **Table SE8.** Comparison of the annotated transporter genes among *Puccinia striiformis* f. sp. *hordei* (*Psh*); *P. striiformis* f. sp. *tritici* (*Psh*); *Blumeria graminis* f. sp. *hordei* (*Bgh*) and *B. graminis* f. sp. *tritici* (*Bgt*). **Table SE9.** The list of fungal genomes and their resources used in this study. **Table SE10.** Summary of orthologues detected in 16 fungal plant pathogens using the OrthoFinder program. **Table SE11.** KEGG pathways annotated using GhostKOALA for *Puccinia striiformis* f. sp. *tritici* (*Pst*) and *P. striiformis* f. sp. *hordei* (*Psh*) isolates. **Table SE11a.** The numbers of conserved, specific and lost genes in *Pst* (93–210) and *Psh* (93TX-2); **Table SE11b.** Annotation of genes of KEGG pathways in *Pst* (93–210); and **Table SE11c.** Annotation of genes of KEGG pathways in *Psh* (93TX-2). **Table SE12.** List of exclusively expressed isolate-specific genes in isolates *Pst* (93–210) and *Psh* (93Tx-2) of *Puccinia striiformis* absent in the genome of the opposite isolate. **Table SE13.** List of exclusively expressed genes in isolates *Pst* (93–210) and *Psh* (93Tx-2) of *Puccinia striiformis* that are present in the genomes of both isolates. (ZIP 1140 kb)

Additional file 3: **Table S1.** The assessment of genome annotations of *Puccinia striiformis* f. sp. *tritici* (*Pst*) and *P. striiformis* f. sp. *hordei* (*Psh*) using the BUSCO program [95]. **Table S2.** Annotated conserved protein-coding and rRNA genes in the mitochondrial genomes of *Puccinia striiformis* f. sp. *tritici* [*Pst* (93–210)] and *P. striiformis* f. sp. *hordei* [*Psh* (93TX-2)]. **Table S3.** Transfer RNA genes in the mitochondria genomes of *Puccinia striiformis* f. sp. *tritici* [*Pst* (93–210)] and *P. striiformis* f. sp. *hordei* [*Psh* (93TX-2)]. **Table S4.** Predicted numbers of variant effects by type in comparison of *Puccinia striiformis* f. sp. *tritici* (*Pst*) and *P. striiformis* f. sp. *hordei* (*Psh*). **Table S5.** Summary functions of genes in isolates *Pst* (93–210) and *Psh* (93TX-2) of *Puccinia striiformis* impacted by mutations and under positive selective. (DOCX 45 kb)

## Abbreviations

*Avr*: Avirulence gene
GO: Gene Ontology
HR: Hyposensitive Reaction
KEGG: Kyoto Encyclopedia of Genes and Genomes
MRCA: Most Recent Common Ancestor
mt: mitochondria
*Psh*: *Puccinia striiformis* f. sp. *hordei*
*Pst*: *Puccinia striiformis* f. sp. *tritici*
SP: Secreted Protein
TE: transposable element
TF: Transcription Factor
