# Supplementary material for: Genomic insights into host adaptation between the wheat stripe rust pathogen (Puccinia striiformis f. sp. tritici) and the barley stripe rust pathogen (Puccinia striiformis f. sp. hordei)
Source: BMC Genomics. 2018 Sep 12;19:664. doi: 10.1186/s12864-018-5041-y (PMC6134786; doi:10.1186/s12864-018-5041-y)
Supplement: Supplementary file 1 — Figure S1. The 17-mer depth distributions of the Illumina reads used to estimate the genome sizes of Pst (93–210) and Psh (93TX-2) isolates of Puccinia striiformis used in this study. Figure S2. Comparisons of functional annotations between wheat-hosted and barley-hosted stripe rust and powdery mildew fungi. Figure S3. Collinearity analysis of syntenic blocks among three Puccinia striiformis isolates. Figure S4. Examples of missing genomic regions by reciprocal mapping of Illumina sequence reads. Figure S5. An overview of a large syntenic region between isolates Ps (93–210) and Psh (93TX-2) of Puccinia striiformis. 4. Figure S6. An overview of the mitochondrial (mt) genomes of isolates Pst (93–210) and Psh (93Tx-2) representing Puccinia striiformis f. sp. tritici (Pst) and P. striiformis f. sp. hordei (Psh), respectively. Figure S7. IGV screenshot showing examples of exclusively expressed genes revealed by reciprocal mapping of Illumina RNA-Seq reads. Figure S8. Genome assembly pipeline used for genome assembly in this study. See details in Methods. (DOCX 4535 kb) [file 12864_2018_5041_MOESM1_ESM.docx]

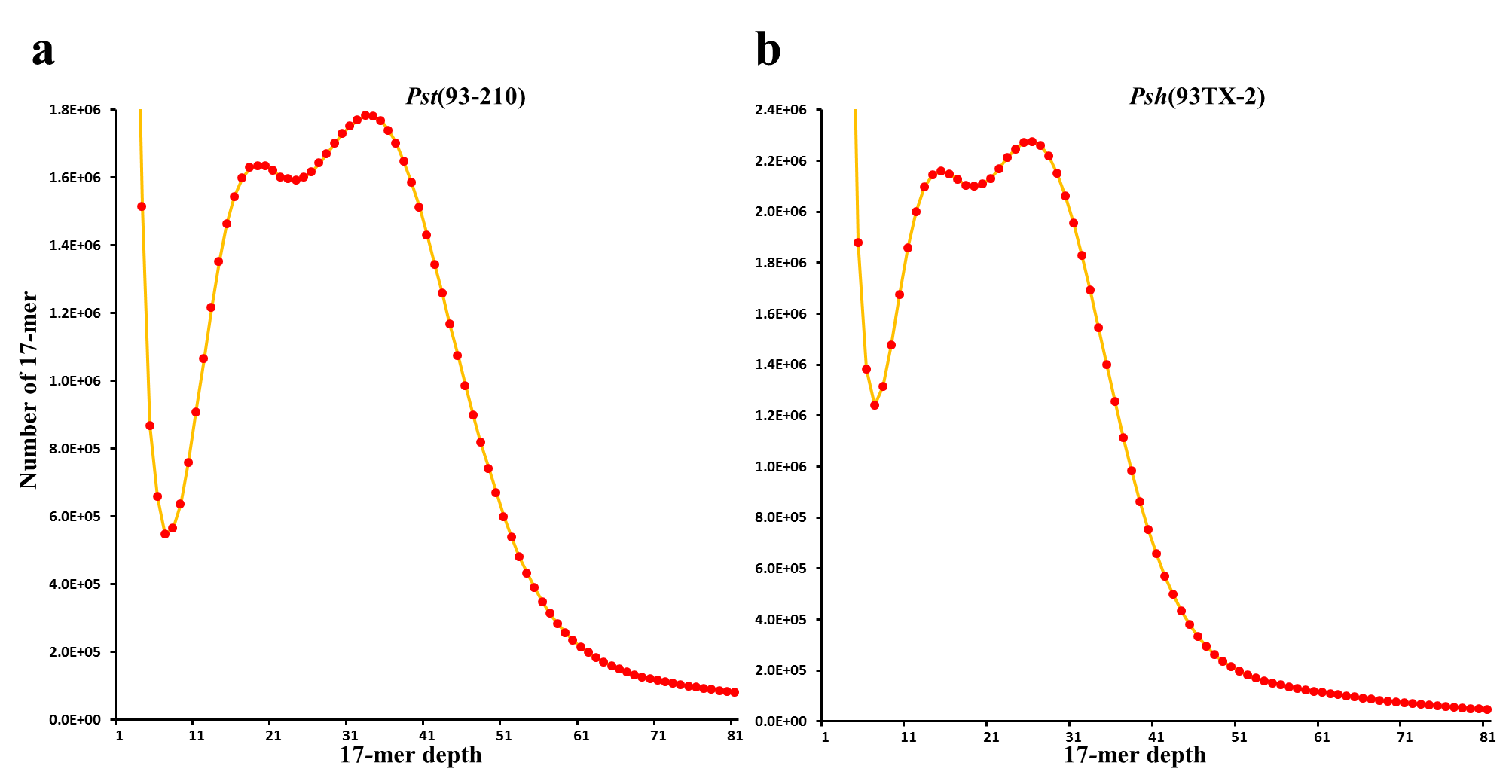


**Fig. S1** The 17-mer depth distributions of the Illumina reads used to estimate the genome sizes of *Pst* (93-210) and *Psh* (93TX-2) isolates of *Puccinia striiformis* used in this study. **a** A total of 103,885,344 17-mer were obtained and two peaks at 19 and 33 were observed for the *Pst* isolate. The estimated genome size was 89,743,503 bp, of which 20,535,944 bp and 69,207,559 bp were estimated to be in heterozygous and homozygous regions, respectively. **b** A total of 166,640,626 17-mer were obtained and two peaks at 15 and 26 were observed for the *Psh* isolate. The estimated genome size was 89,709,453 bp, of which 18,880,342 bp and 70,829,111 bp were estimated to be in heterozygous and homozygous regions, respectively.


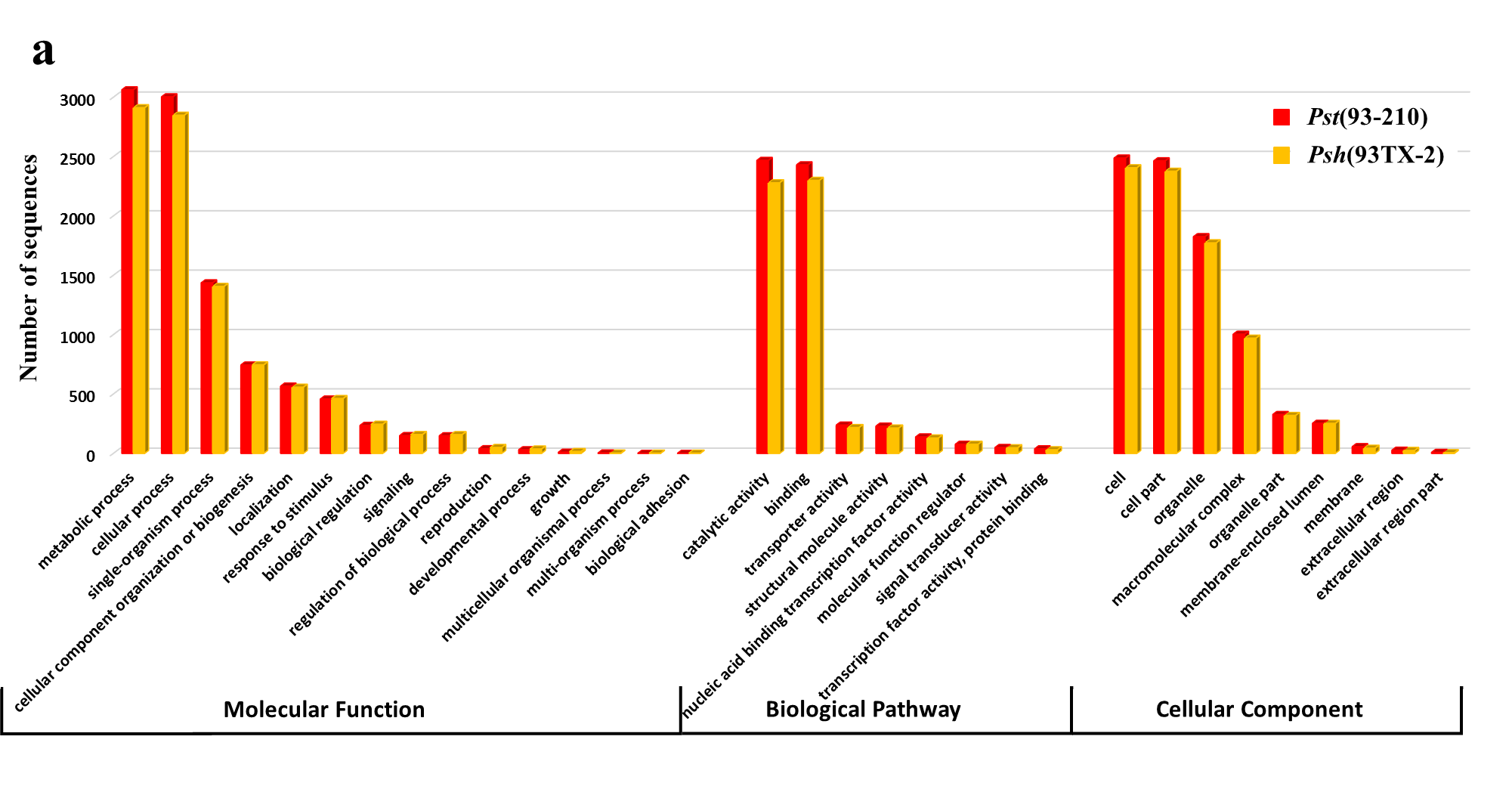


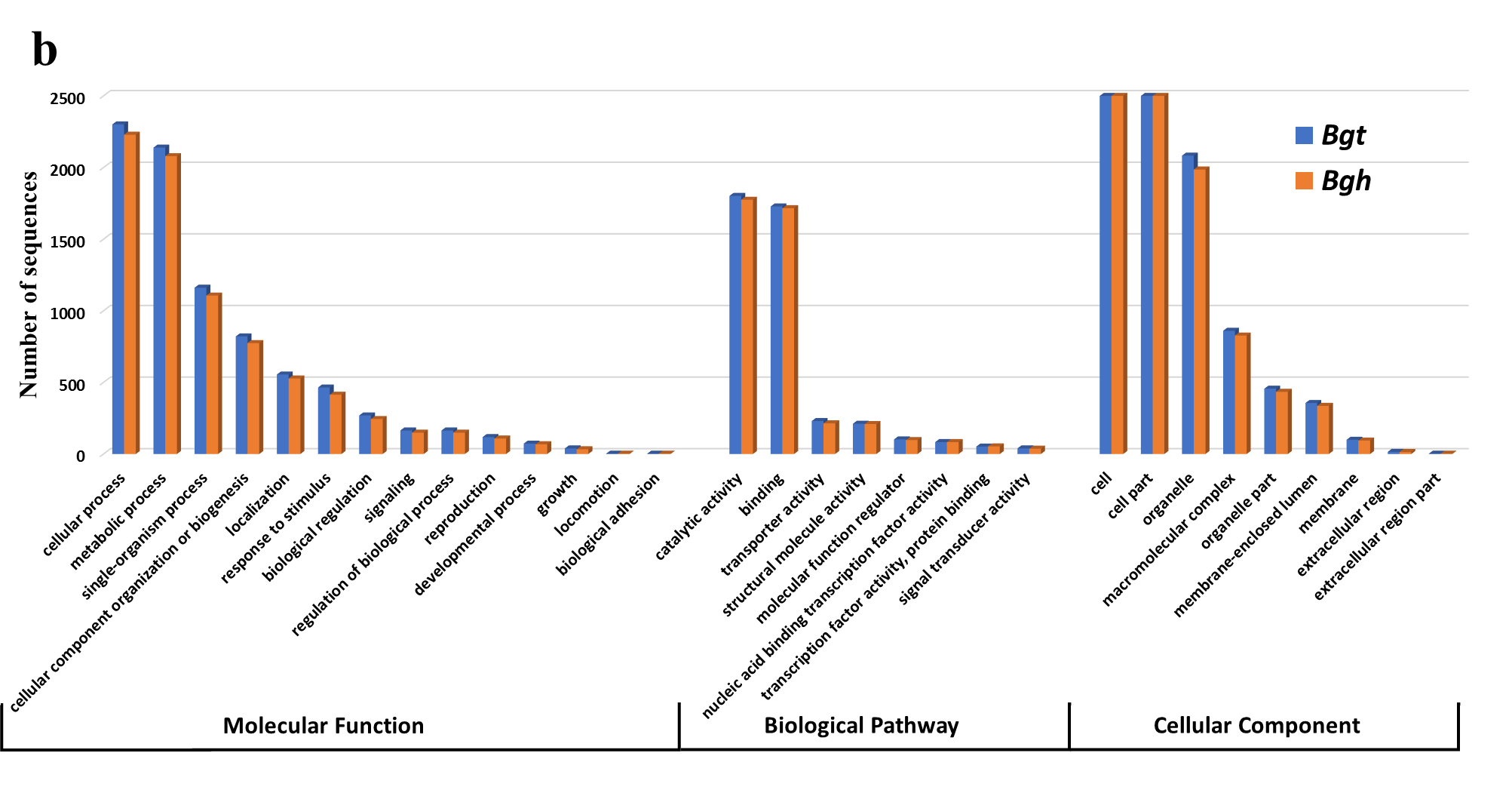


**Fig. S2** Comparisons of functional annotations between wheat-hosted and barley-hosted stripe rust and powdery mildew fungi. **a** Comparison between wheat-hosted *Pst* (93-210) and barley-hosted *Psh* (93TX-2) isolates. **b** Comparison between wheat-hosted *Bgt* and barley-hosted *Bgh* isolates. The *Pst* and *Psh* data were from this study and the *Bgt* and *Bgh* data were from Wicker et al. 2013 and Spanu et al. 2010. Functional annotations were summarized using program BLAST2GO v4.1.9. *Pst*, *Puccinia striiformis* f. sp. *tritici*; *Psh*, *P. striiformis* f. sp. *hordei*; *Bgt*, *Blumeria graminis* f. sp. *tritici*; and *Bgh*, *B. graminis* f. sp. *hordei*.


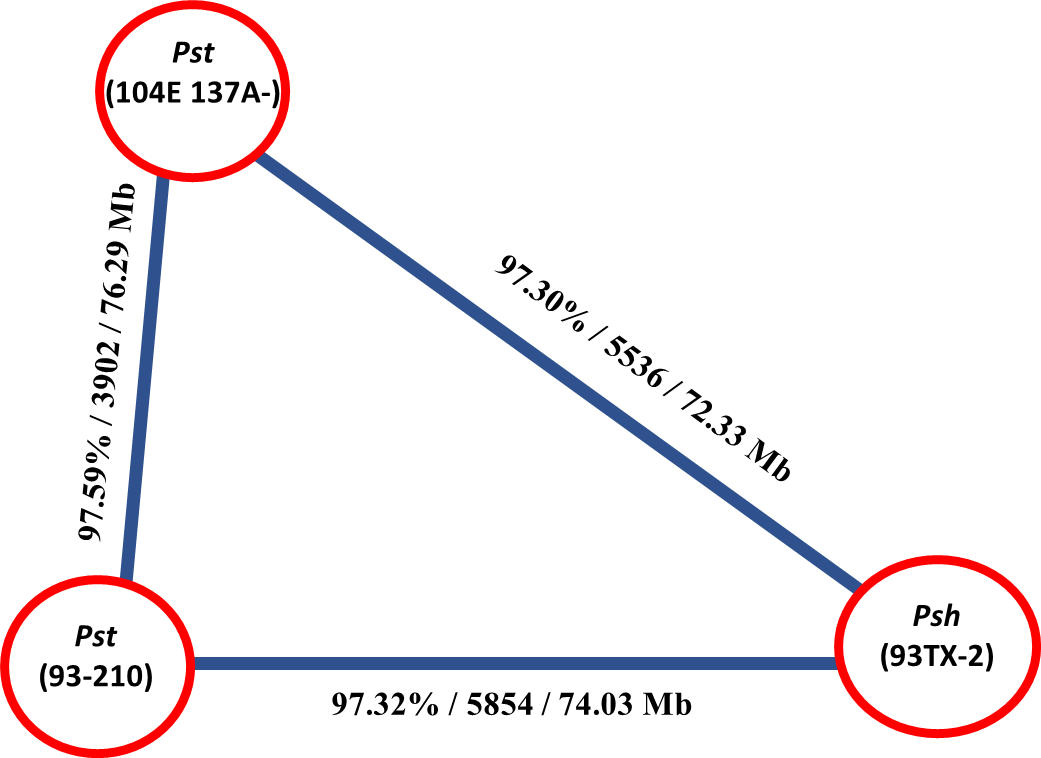


**Fig. S3** Collinearity analysis of syntenic blocks among three *Puccinia striiformis* isolates. Three numbers along each triangle edge are average identity / total number / total size of syntenic blocks. The identity between two *Pst* isolates is higher than identities between *Pst* and *Psh* isolates. Syntenic blocks were identified using MUMmer v3.23.


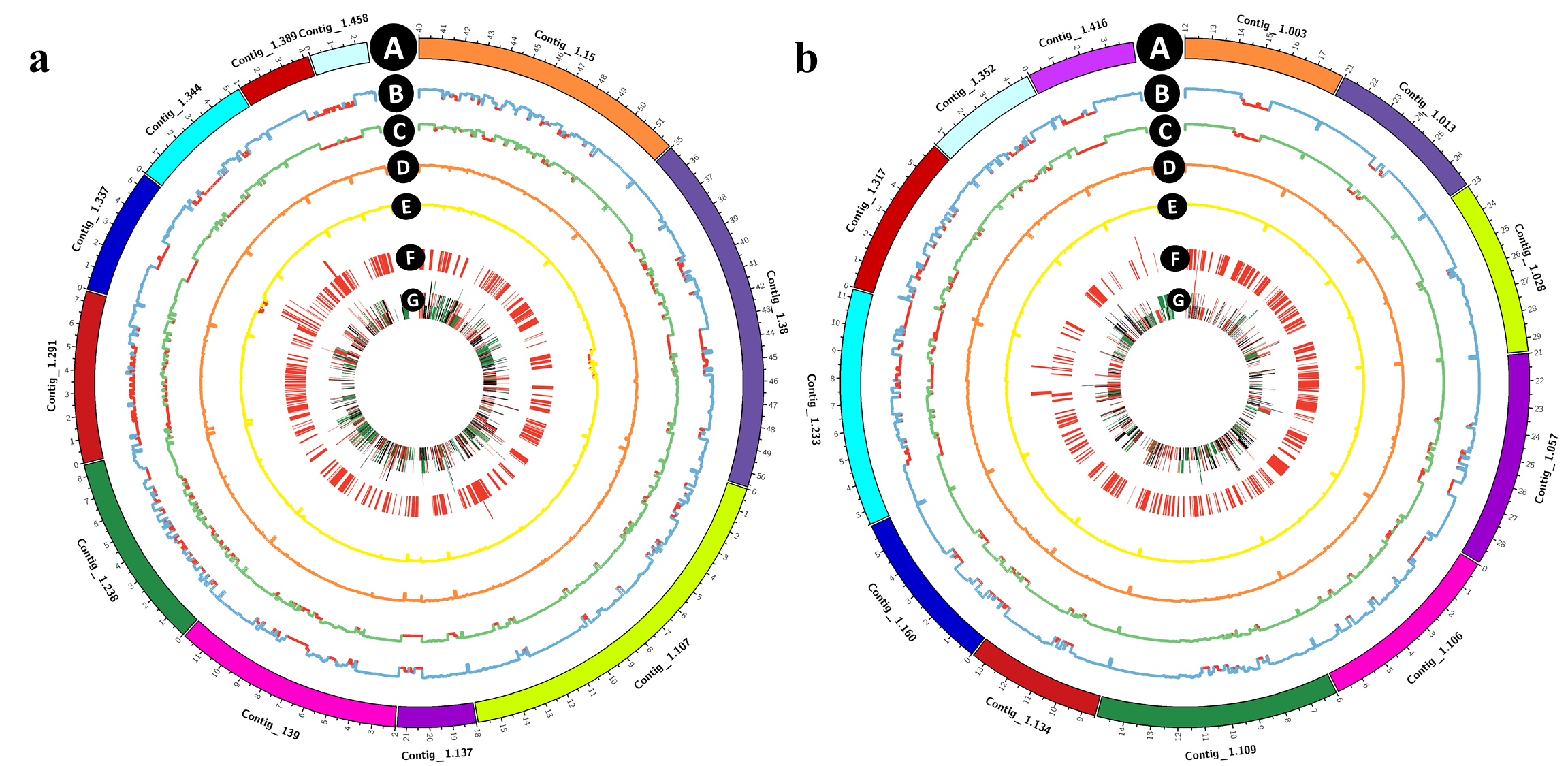


**
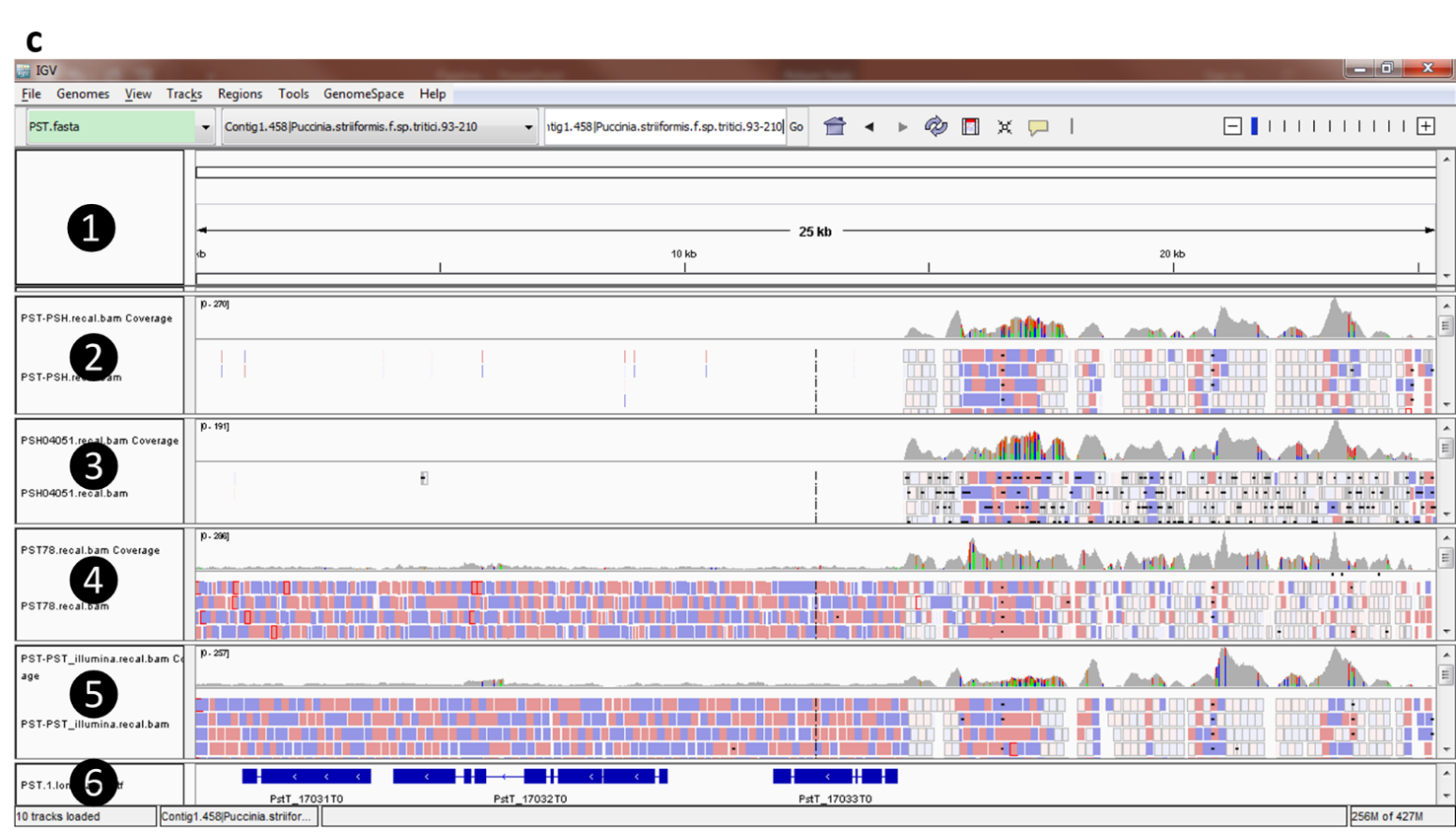
**

**
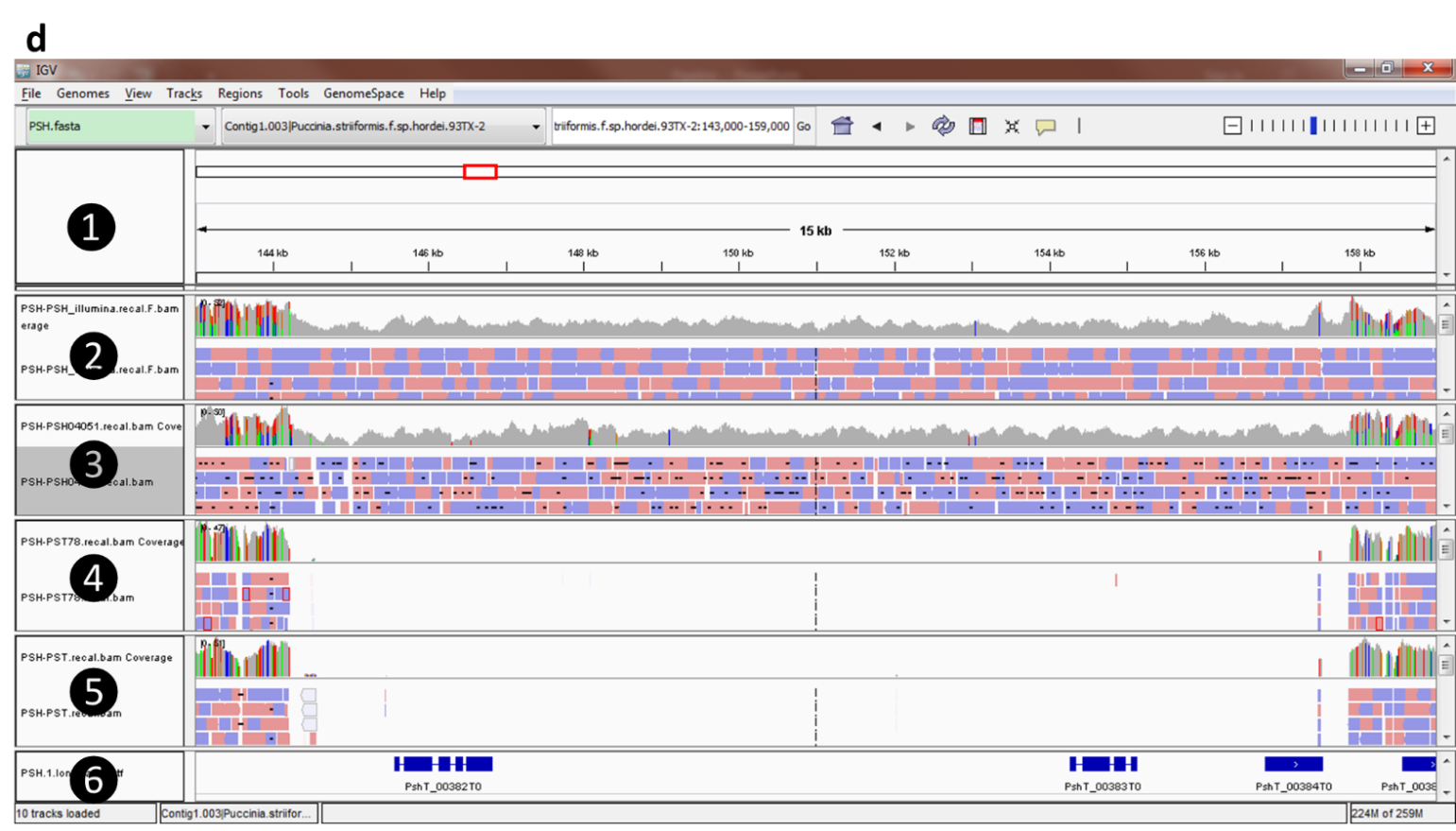
**

**Fig. S4** Examples of missing genomic regions by reciprocal mapping of Illumina sequence reads. **a** Examples of *Puccinia striiformis* f. sp. *tritici* (*Pst*) regions that are present in two *Pst* isolates but absent in two *Psh* isolates. In these circles, **A** denotes contigs of the reference genome *Pst* (93-210); **B-E** histograms of mapping coverage when genomic sequencing reads of *Psh* (93TX-2), *Psh* (04-051), *Pst* (93-210), and 2K-41-Yr9 were mapped; **F** each red bar represents one gene; and **G** each bar represents one transposable element. **b** Examples of *P. striiformis* f. sp. *hordei* (*Psh*) regions that are present in two *Psh* isolates but absent in two *Pst* isolates. **A** Denotes contigs of the reference genome *Psh* (93TX-2); **B-E** histograms of mapping coverage when genomic sequencing reads of *Pst* (93-210), 2K-41-Yr9, *Psh* (93TX-2), and *Psh* (04-051) were mapped; and **F** and **G** are the same as in **a**. A 1000bp window size was used for coverage. **c** and **d** IGV screenshot of reciprocal mapping of Illumina reads showing the missing regions and genes in one isolate but present in the other. Track❶, the reference genome. Track❷, mapping of *Psh* (93TX-2) sequence reads. Track❸, mapping of *Psh* (04-051) reads. Track❹, mapping of *Pst* (93-210) reads. Track❺, mapping of 2K-41-Yr9 reads. Track❻, gene structure.


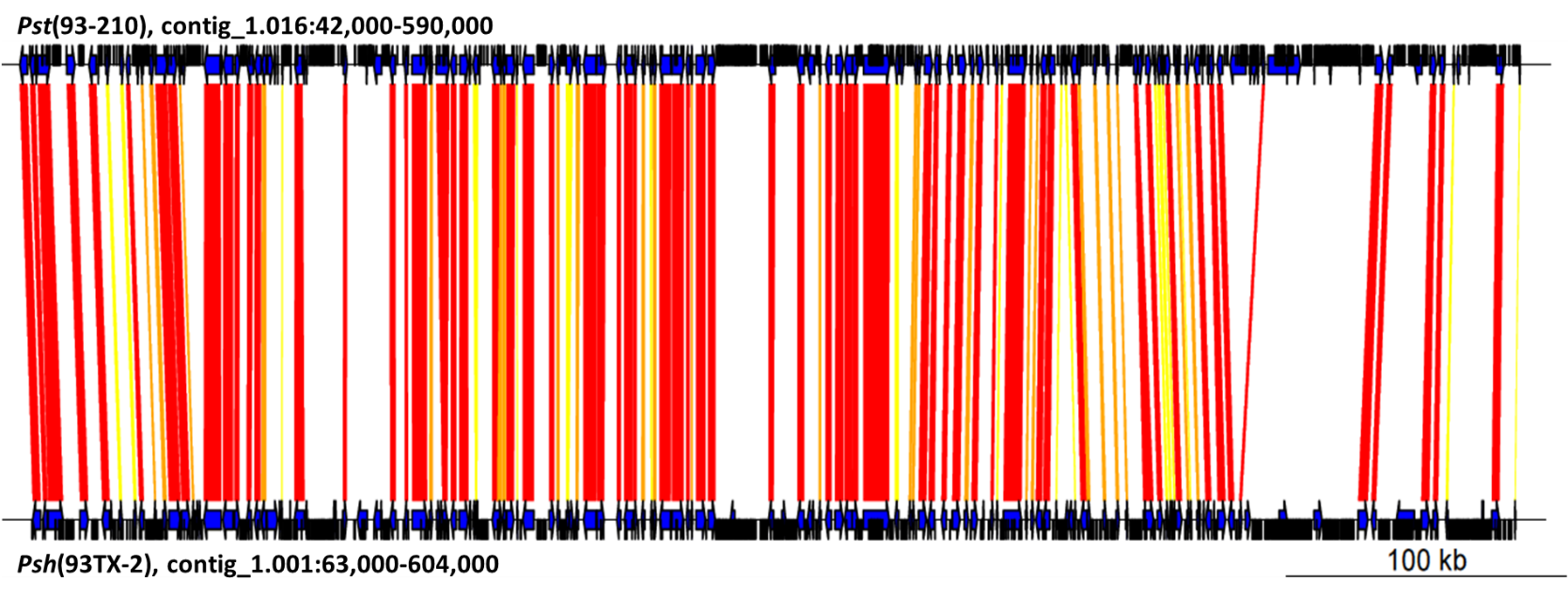


**Fig. S5** An overview of a large syntenic region between isolates *Pst* (93-210) and *Psh* (93TX-2) of *Puccinia striiformis*. See the legends in **Fig. 4**.


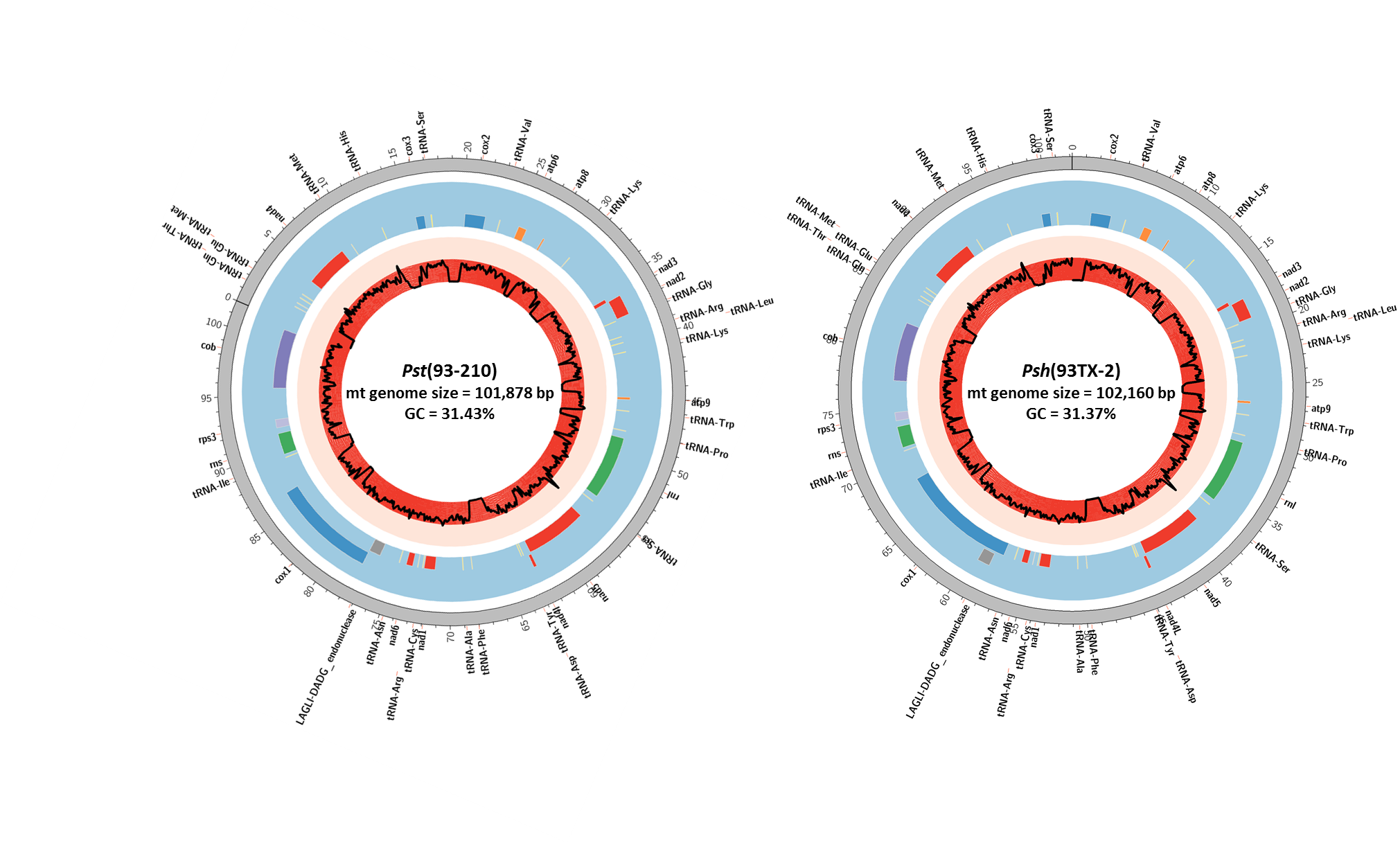


**Fig. S6** An overview of the mitochondrial (mt) genomes of isolates *Pst* (93-210) and *Psh* (93Tx-2) representing *Puccinia striiformis* f. sp. *tritici* (*Pst*) and *P. striiformis* f. sp. *hordei* (*Psh*), respectively. The outer layer ideogram represents physical position of the mt chromosome, with a unit of 1 kb. Tiles in the middle layer represent genes. Yellow: tRNA-coding genes; red: NADH dehydrogenase-coding gene family; orange: ATP synthase-coding gene family; blue: cytochrome c oxidase-coding gene family; green: subunit ribosomal RNA (*rnl* and *rns*); purple: cytochrome b gene. The black line in the inner layer shows the distribution of GC content, with the inner boundary of red background 0% GC content, middle boundary 50%, and outer boundary of pink background 100% GC content.


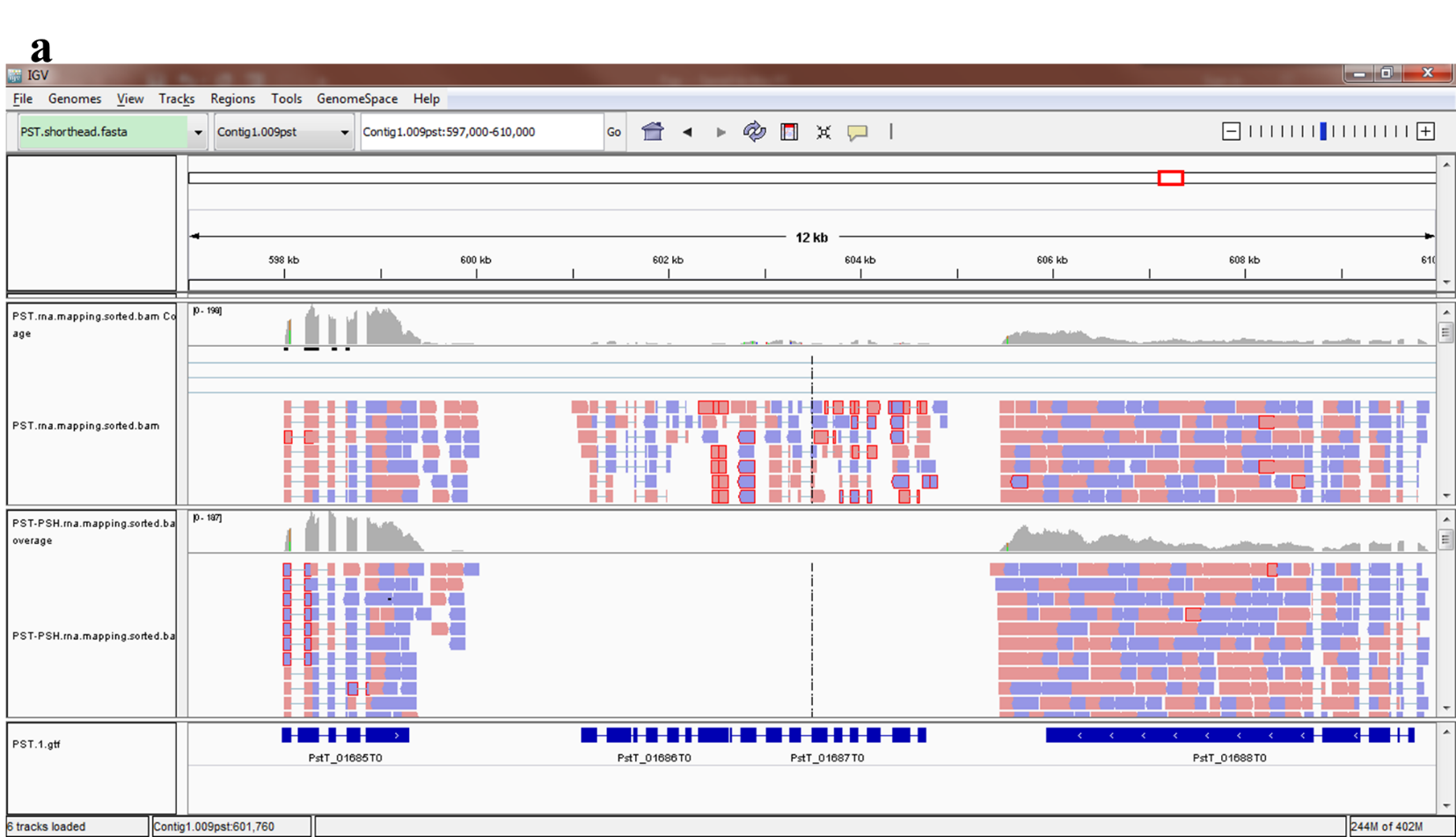


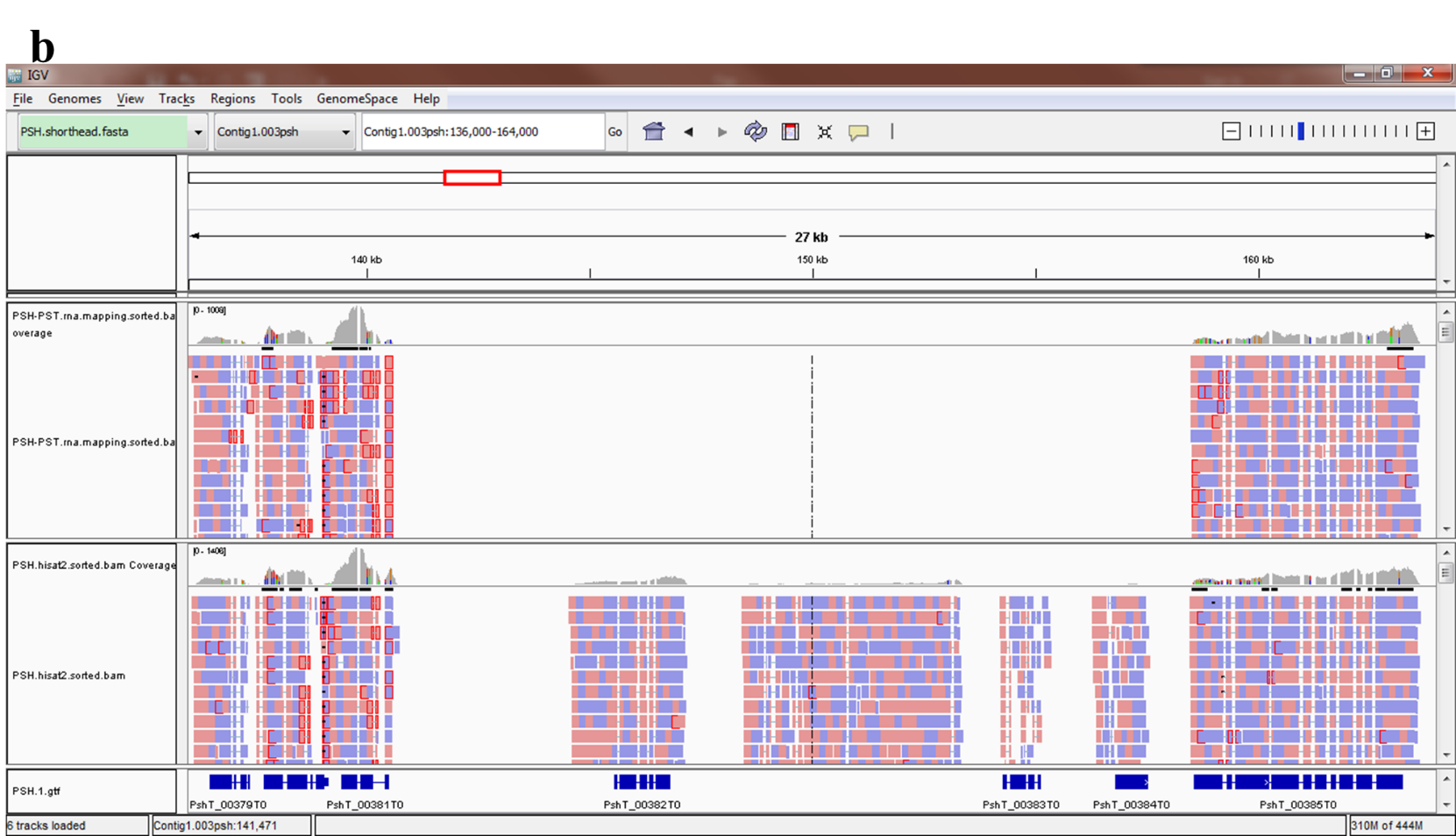


**Fig. S7** IGV screenshot showing examples of exclusively expressed genes revealed by reciprocal mapping of Illumina RNA-Seq reads. **a** Two *Pst* (93-210) genes (*PstG_01686T0* and *PstG_01687T0*) are expressed in *Pst* (93-210), but not expressed in *Psh* (93TX-2). **b** Three *Psh* (93TX-2) genes (*PshG_00382T0*, *PshG_00383T0* and *PshG_00384T0*) are expressed in *Psh* (93TX-2) but not expressed in *Pst* (93-210). See legends of **Fig. S4**, except that the sequencing reads are RNA-Seq reads.


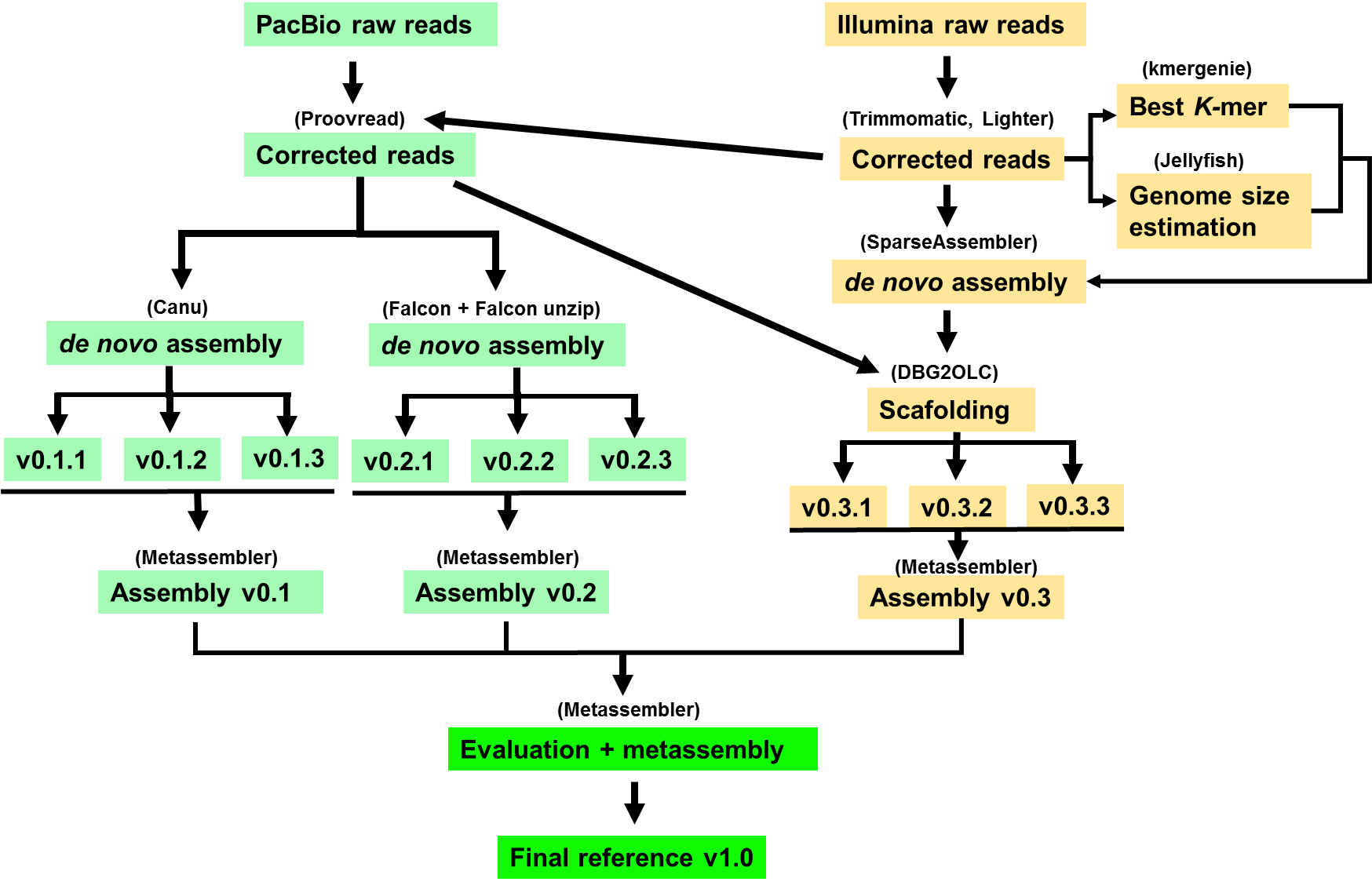


**Fig. S8** Pipeline used for genome assembly in this study.
