# Supplementary material for: Genomic insights into host adaptation between the wheat stripe rust pathogen (Puccinia striiformis f. sp. tritici) and the barley stripe rust pathogen (Puccinia striiformis f. sp. hordei)
Source: BMC Genomics. 2018 Sep 12;19:664. doi: 10.1186/s12864-018-5041-y (PMC6134786; doi:10.1186/s12864-018-5041-y)
Supplement: Supplementary file 3 — Table S1. The assessment of genome annotations of Puccinia striiformis f. sp. tritici (Pst) and P. striiformis f. sp. hordei (Psh) using the BUSCO program [95]. Table S2. Annotated conserved protein-coding and rRNA genes in the mitochondrial genomes of Puccinia striiformis f. sp. tritici [Pst (93–210)] and P. striiformis f. sp. hordei [Psh (93TX-2)]. Table S3. Transfer RNA genes in the mitochondria genomes of Puccinia striiformis f. sp. tritici [Pst (93–210)] and P. striiformis f. sp. hordei [Psh (93TX-2)]. Table S4. Predicted numbers of variant effects by type in comparison of Puccinia striiformis f. sp. tritici (Pst) and P. striiformis f. sp. hordei (Psh). Table S5. Summary functions of genes in isolates Pst (93–210) and Psh (93TX-2) of Puccinia striiformis impacted by mutations and under positive selective. (DOCX 45 kb) [file 12864_2018_5041_MOESM3_ESM.docx]

**Table S1** The assessment of genome annotations of Puccinia striiformis f. sp. tritici (Pst) and P. striiformis f. sp. hordei (Psh) using the BUSCO program

|  | ***Pst* (93-210)** | |  | ***Psh* (93TX-2)** | |  | ***Pst* 104E 137A-** | |
| --- | --- | --- | --- | --- | --- | --- | --- | --- |
|  | **Proteins** | **Percentage** |  | **Proteins** | **Percentage** |  | **Proteins** | **Percentage** |
| Total BUSCO groups searched | 1335 | 100.00% |  | 1335 | 100.00% |  | 1335 | 100% |
|  |  |  |  |  |  |  |  |  |
| Complete single-copy BUSCOs | 1015 | 76.00% |  | 1018 | 76.30% |  | 1121 | 84.00% |
|  |  |  |  |  |  |  |  |  |
| Complete duplicated BUSCOs | 119 | 8.90% |  | 103 | 7.70% |  | 133 | 10.00% |
|  |  |  |  |  |  |  |  |  |
| Fragmented BUSCOs | 136 | 10.20% |  | 120 | 9.00% |  | 48 | 3.60% |
|  |  |  |  |  |  |  |  |  |
| Missing BUSCOs | 65 | 4.90% |  | 94 | 7.00% |  | 33 | 2.40% |

**Table S2** Annotated conserved protein-coding and rRNA genes in the mitochondrial genomes of Puccinia striiformis f. sp. tritici [Pst (93-210)] and P. striiformis f. sp. hordei [Psh (93TX-2)]

| **Gene** | **Start** | **End** | **Length (bp)** | **Exon length (bp)** | **Strand** |
| --- | --- | --- | --- | --- | --- |
| ***Pst* (93-210)** |  |  |  |  |  |
| *nad4* | 8237 | 4308 | 3930 | 1437 | - |
| *cox3* | 16452 | 15649 | 804 | 804 | - |
| *cox2* | 22053 | 20184 | 1870 | 753 | - |
| *atp6* | 25982 | 25212 | 771 | 771 | - |
| *atp8* | 27823 | 27677 | 147 | 147 | - |
| *nad3* | 35967 | 35635 | 333 | 333 | - |
| *nad2* | 37714 | 35990 | 1725 | 1329 | - |
| *atp9* | 45139 | 44918 | 222 | 222 | - |
| *rnl* | 54534 | 48701 | 5834 | 2573 | - |
| *nad5* | 62510 | 56809 | 5702 | 1797 | - |
| *nad4L* | 62775 | 62515 | 261 | 261 | - |
| *nad1* | 72410 | 71418 | 993 | 993 | - |
| *nad6* | 74071 | 73472 | 600 | 600 | - |
| *LAGLI-DADG_endonuclease* | 77647 | 76577 | 1071 | 1071 | - |
| *cox1* | 86473 | 75759 | 10715 | 1533 | - |
| *rns* | 91537 | 89597 | 1941 | 1941 | - |
| *rps3* | 92837 | 92090 | 748 | 558 | - |
| *cob* | 101164 | 95744 | 5421 | 1158 | - |
|  |  |  |  |  |  |
| ***Psh* (93TX-2)** |  |  |  |  |  |
| cox2 | 3598 | 1732 | 1867 | 753 | - |
| atp6 | 7527 | 6757 | 771 | 771 | - |
| atp8 | 9372 | 9226 | 147 | 147 | - |
| nad3 | 17524 | 17180 | 345 | 345 | - |
| nad2 | 19259 | 17535 | 1725 | 1329 | - |
| atp9 | 26745 | 26524 | 222 | 222 | - |
| rnl | 36236 | 30373 | 5864 | 2589 | - |
| nad5 | 44221 | 38516 | 5706 | 1797 | - |
| nad4L | 44486 | 44226 | 261 | 261 | - |
| nad1 | 54114 | 53122 | 993 | 927 | - |
| nad6 | 55778 | 55179 | 600 | 600 | - |
| *LAGLI-DADG_endonuclease* | 59354 | 58284 | 1071 | 1071 | - |
| cox1 | 68196 | 57466 | 10731 | 1533 | - |
| rns | 73283 | 71319 | 1965 | 1215 | - |
| rps3 | 74590 | 73838 | 753 | 570 | - |
| cob | 82927 | 77505 | 5423 | 1158 | - |
| nad4 | 91944 | 88013 | 3932 | 1434 | - |
| cox3 | 100154 | 99351 | 804 | 804 | - |

**Table S3** Transfer RNA genes in the mitochondria genomes of Puccinia striiformis f. sp. tritici [Pst (93-210)] and P. striiformis f. sp. hordei [Psh (93TX-2)]

| **tRNA** | **Codon usage** | **Start** | **End** | **Length (bp)** |
| --- | --- | --- | --- | --- |
| ***Pst* (93-210)** |  |  |  |  |
| tRNA-Ala | GCA | 68884 | 68814 | 71 |
| tRNA-Arg | CGA | 39355 | 39285 | 71 |
|  | AGA | 73047 | 72977 | 71 |
| tRNA-Asn | AAC | 74764 | 74693 | 72 |
| tRNA-Asp | GAC | 63230 | 63159 | 72 |
| tRNA-Cys | UGC | 72598 | 72526 | 73 |
| tRNA-Gln | CAA | 1630 | 1559 | 72 |
| tRNA-Glu | GAA | 2544 | 2473 | 72 |
| tRNA-Gly | GGA | 37883 | 37812 | 72 |
| tRNA-His | CAC | 12480 | 12407 | 74 |
| tRNA-Ile | AUC | 89303 | 89231 | 73 |
| tRNA-Leu | CUA | 39916 | 39834 | 83 |
| tRNA-Lys | AAG | 30693 | 30622 | 72 |
|  | AAA | 40796 | 40724 | 73 |
| tRNA-Met | AUG | 2918 | 2847 | 72 |
|  | AUG | 9318 | 9248 | 71 |
| tRNA-Phe | UUC | 68046 | 67974 | 73 |
| tRNA-Pro | CCA | 48067 | 47996 | 72 |
| tRNA-Ser | UCA | 17137 | 17054 | 84 |
|  | AGC | 55149 | 55066 | 84 |
| tRNA-Thr | ACA | 2012 | 1939 | 74 |
| tRNA-Trp | UGA | 46235 | 46164 | 72 |
| tRNA-Tyr | UAC | 63425 | 63343 | 83 |
| tRNA-Val | GUA | 23439 | 23368 | 72 |
|  |  |  |  |  |
| ***Psh* (93TX-2)** |  |  |  |  |
| tRNA-Ala | GCA | 50590 | 50520 | 71 |
| tRNA-Arg | CGA | 20897 | 20827 | 71 |
|  | AGA | 54753 | 54683 | 71 |
| tRNA-Asn | AAC | 56471 | 56400 | 72 |
| tRNA-Asp | GAC | 44940 | 44869 | 72 |
| tRNA-Cys | UGC | 54303 | 54231 | 73 |
| tRNA-Gln | CAA | 85333 | 85262 | 72 |
| tRNA-Glu | GAA | 86244 | 86173 | 72 |
| tRNA-Gly | GGA | 19428 | 19357 | 72 |
| tRNA-His | CAC | 96184 | 96111 | 74 |
| tRNA-Ile | AUC | 71025 | 70953 | 73 |
| tRNA-Leu | CUA | 21459 | 21377 | 83 |
| tRNA-Lys | AAG | 12243 | 12172 | 72 |
|  | AAA | 22366 | 22294 | 73 |
| tRNA-Met | AUG | 86618 | 86547 | 72 |
|  | AUG | 93022 | 92952 | 71 |
| tRNA-Phe | UUC | 49749 | 49677 | 73 |
| tRNA-Pro | CCA | 29742 | 29671 | 72 |
| tRNA-Ser | AGC | 36852 | 36769 | 84 |
|  | UCA | 100841 | 100758 | 84 |
| tRNA-Thr | ACA | 85712 | 85639 | 74 |
| tRNA-Trp | UGA | 27850 | 27779 | 72 |
| tRNA-Tyr | UAC | 45134 | 45052 | 83 |
| tRNA-Val | GUA | 4986 | 4915 | 72 |

**Table S4** Predicted numbers of variant effects by type in comparison of Puccinia striiformis f. sp. tritici (Pst) and P. striiformis f. sp. hordei (Psh)

| **Effect ontology** | ***Pst* (93-210)** | |  | ***Psh* (93TX-2)** | |
| --- | --- | --- | --- | --- | --- |
|  | ***Pst* (93-210)** | ***Psh* (93TX-2)** |  | ***Pst* (93-210)** | ***Psh* (93TX-2)** |
| **SNPs** |  |  |  |  |  |
| 3_prime_UTR | 631 | 795 |  | 802 | 689 |
| downstream_gene | 568714 | 689340 |  | 762545 | 634342 |
| initiator_codon | 16 | 23 |  | 22 | 19 |
| intergenic_region | 309598 | 378804 |  | 401370 | 332492 |
| intron | 81131 | 95308 |  | 107079 | 88903 |
| missense | 58667 | 69957 |  | 80226 | 65255 |
| splice_acceptor* | 446 | 491 |  | 549 | 493 |
| splice_donor* | 495 | 533 |  | 615 | 526 |
| splice_region | 11260 | 13108 |  | 13850 | 11595 |
| start_lost* | 114 | 127 |  | 149 | 122 |
| stop_gained* | 1404 | 1673 |  | 1935 | 1685 |
| stop_lost* | 206 | 234 |  | 307 | 268 |
| stop_retained | 143 | 175 |  | 195 | 167 |
| synonymous | 69617 | 84179 |  | 95170 | 75438 |
| upstream_gene | 552651 | 668323 |  | 744327 | 612596 |
|  |  |  |  |  |  |
| **InDels** |  |  |  |  |  |
| 3_prime_UTR | 298 | 275 |  | 240 | 226 |
| bidirectional_gene_fusion | 1 | 2 |  | 0 | 1 |
| conservative_inframe_deletion | 335 | 421 |  | 511 | 433 |
| conservative_inframe_insertion | 819 | 893 |  | 923 | 827 |
| disruptive_inframe_deletion | 677 | 844 |  | 914 | 768 |
| disruptive_inframe_insertion | 844 | 913 |  | 1066 | 972 |
| downstream_gene | 182770 | 181678 |  | 197092 | 193061 |
| exon_loss* | 3 | 5 |  | 3 | 2 |
| frameshift* | 22751 | 20253 |  | 21768 | 23278 |
| intergenic_region | 92630 | 93115 |  | 98216 | 94862 |
| intragenic | 1 | 1 |  | 0 | 0 |
| intron | 38073 | 37720 |  | 40447 | 39657 |
| missense | 328 | 243 |  | 291 | 363 |
| non_coding_transcript | 1 | 1 |  | 2 | 1 |
| splice_acceptor* | 457 | 418 |  | 424 | 458 |
| splice_donor* | 389 | 335 |  | 324 | 373 |
| splice_region | 5690 | 5297 |  | 5602 | 5717 |
| start_lost* | 60 | 58 |  | 76 | 70 |
| stop_gained* | 465 | 413 |  | 408 | 441 |
| stop_lost* | 120 | 105 |  | 129 | 130 |
| synonymous | 138 | 106 |  | 133 | 147 |
| upstream_gene | 181028 | 178948 |  | 194215 | 189806 |

* Effects of variations that have high predicted impacts on protein functions or phenotypes.

**Table S5** Summary functions of genes in isolates Pst (93-210) and Psh (93TX-2) of Puccinia striiformis impacted by mutations and under positive selective

|  | **Mutations** | |  | **dN/dS** | |
| --- | --- | --- | --- | --- | --- |
|  | ***Pst* (93-210)** | ***Psh* (93TX-2)** |  | **Purifying selection (%)** | **Positive selection (%)** |
| Total genes | 5658 | 5908 |  | 3215 | 1271 |
| CAZymes | 167 | 178 |  | 61(1.89) | 9(0.70) |
| Cytochrome P450 | 7 | 8 |  | 1(0.03) | 0(0.00) |
| Expressed genes | 3066 | 3117 |  | 2182(67.86) | 614(48.30) |
| Genes involved in pathogen-host interaction | 333 | 326 |  | 229(7.12) | 31(2.4) |
| Proteases | 476 | 500 |  | 332(10.32) | 66(5.19) |
| Secondary metabolites | 19 | 14 |  | 4(0.12) | 1(0.07) |
| Secreted proteins | 474 | 534 |  | 320(9.95) | 146(11.48) |
| Transcription factors | 33 | 23 |  | 19(0.59) | 5(0.39) |
| Transporters | 157 | 167 |  | 121(3.76) | 16(1.25) |

**Table S6** Statistics of the Pst (93-210) intermediate assemblies

| **Canu** | **Raw + correction, trimming, assembly** | **Trimmed +**  **assembly** | **Untrimmed + trimming, assembly** | **Meta-assembly** |
| --- | --- | --- | --- | --- |
| Version | 0.1.1 | 0.1.2 | 0.1.3 | 0.1 |
| No. of contigs | 1,343 | 1,867 | 1,652 | 1,132 |
| No. (%) of contigs  > 100kb | 278 (20.7%) | 327 (17.5%) | 289 (17.5%) | 287 (25.4%) |
| No. of contigs > 1Mb | 0 | 0 | 2 | 2 |
| Total contig size (Mb) | 97 | 132 | 130 | 115 |
| N50 (kb) | 127 | 133 | 153 | 200 |
| Mean contig size (kb) | 72 | 70 | 79 | 102 |
| GC (%) | 44.34 | 43.89 | 43.96 | 44.39 |
|  |  |  |  |  |
| Falcon + Falcon unzip | p_ctg | h_ctg |  |  |
| version | 0.2 | 0.2 |  |  |
| No. of contigs | 533 | 885 |  |  |
| No. (%) of contigs  > 100kb | 241 (45.2%) | 174 (19.7%) |  |  |
| No. of contigs > 1Mb | 3 | 0 |  |  |
| Total contig size (Mb) | 85 | 55 |  |  |
| N50 (kb) | 290 | 110 |  |  |
| Mean contig size (kb) | 160 | 62 |  |  |
| GC (%) | 44.35 | 44.36 |  |  |
|  |  |  |  |  |
| SparseAssembler + DBG2OLC | Raw | Trimmed | Untrimmed | Meta-assembly |
| version | 0.3.1 | 0.3.2 | 0.3.3 | 0.3 |
| No. of contigs | 1,255 | 1,317 | 1,208 | 975 |
| No. (%) of contigs  > 100kb | 368 (29.3%) | 378 (28.7%) | 394 (32.6%) | 394 (40.4%) |
| No. of contigs > 1Mb | 0 | 0 | 0 | 0 |
| Total contig size (Mb) | 114 | 116 | 119 | 114 |
| N50 (kb) | 157 | 156 | 170 | 182 |
| Mean contig size (kb) | 91 | 88 | 98 | 117 |
| GC (%) | 44.36 | 44.38 | 44.39 | 44.39 |

**Table S7** Statistics of Psh (93TX-2) intermediate assemblies

| **Canu** | **Raw + correction, trimming, assembly** | **Trimmed +**  **assembly** | **Untrimmed +**  **trimming, assembly** | **Meta-assembly** |
| --- | --- | --- | --- | --- |
| Version | 0.1.1 | 0.1.2 | 0.1.3 | 0.1 |
| No. of contigs | 1,261 | 1,809 | 1,543 | 1,033 |
| No. (%) of contigs  > 100kb | 254 (20.1%) | 319 (17.6%) | 301 (19.5%) | 299 (28.9%) |
| No. of contigs > 1Mb | 2 | 0 | 3 | 3 |
| Total contig size (Mb) | 87 | 123 | 122 | 107 |
| N50 (kb) | 119 | 122 | 146 | 187 |
| Mean contig size (kb) | 69 | 68 | 79 | 104 |
| GC (%) | 44.44 | 43.77 | 43.89 | 44.36 |
|  |  |  |  |  |
| Falcon + Falcon unzip | p_ctg | h_ctg |  |  |
| version | 0.2 | 0.2 |  |  |
| No. of contigs | 598 | 951 |  |  |
| No. (%) of contigs  > 100kb | 261 (43.6%) | 93 (9.8%) |  |  |
| No. of contigs > 1Mb | 0 | 0 |  |  |
| Total contig size (Mb) | 77 | 45 |  |  |
| N50 (kb) | 216 | 66 |  |  |
| Mean contig size (kb) | 130 | 47 |  |  |
| GC (%) | 44.33 | 44.37 |  |  |
|  |  |  |  |  |
| SparseAssembler + DBG2OLC | Raw | Trimmed | Untrimmed | Meta-assembly |
| version | 0.3.1 | 0.3.2 | 0.3.3 | 0.3 |
| No. of contigs | 1,211 | 1,206 | 1,615 | 850 |
| No. (%) of contigs  > 100kb | 362 (29.9%) | 365 (30.3%) | 364 (22.5%) | 364 (42.8%) |
| No. of contigs > 1Mb | 0 | 0 | 1 | 1 |
| Total contig size (Mb) | 109 | 110 | 121 | 107 |
| N50 (kb) | 170 | 162 | 181 | 208 |
| Mean contig size (kb) | 90 | 91 | 75 | 126 |
| GC (%) | 44.35 | 44.37 | 44.87 | 44.35 |
